# Bioinformatics identification of key genes correlating NOD1 and Endoplasmic Reticulum stress in Hepatitis B virus-induced acute liver failure

**DOI:** 10.1038/s41598-025-19813-x

**Published:** 2025-10-14

**Authors:** Fuexue Deng, Wei Jiang, Ning Wang, Yuchao Wu, Jing Xu, Rongrong Hou, Fang Jia

**Affiliations:** 1 Department of Cardiology, The Second Affiliated Hospital of Xi’an Jiaotong University, Xi’an, 710004 Shaanxi China; 2 Department of Endocrinology and Metabolism, The Second Affiliated Hospital of Xi’an Jiaotong University, Xi’an, 710004 Shaanxi China

**Keywords:** Endoplasmic reticulum stress, Hepatitis b virus-associated acute liver failure, Immune infiltration, Key genes, Hepatitis, Liver diseases, Prognostic markers

## Abstract

Endoplasmic reticulum stress (ERS) has been implicated in a range of biological processes, yet its specific involvement in Hepatitis B virus-associated acute liver failure (HBV-ALF) remains poorly understood. This study aimed to identify key ERS-related genes (ERGs) and elucidate their underlying mechanisms in HBV-ALF. Publicly available HBV-ALF-related datasets (GSE38941, GSE62029) and ERGs were analyzed. Intersection genes were determined by overlapping differentially expressed genes from both datasets with ERGs, and genes showing strong correlation with NOD1 were selected as candidates. The BottleNeck algorithm in the Cytohubba plugin and machine learning-based screening were subsequently applied to refine key gene selection. Diagnostic performance was assessed using ROC curves, while a nomogram was constructed to evaluate the predictive value for HBV-ALF. Functional enrichment and immune-related analyses were also conducted on the identified key genes. The results revealed that among 5,699 intersection genes, 265 overlapped with ERGs, resulting in 97 key intersection genes. Of these, 86 showed strong correlation with NOD1. From the top 20 genes identified by the BottleNeck algorithm, five key genes—SEL1L, DNAJB9, DERL3, NOD1, and CFTR—were ultimately selected through machine learning. ROC analysis demonstrated that all five genes exhibited high diagnostic accuracy, with AUC values exceeding 0.8, effectively distinguishing HBV-ALF samples from normal controls. The nomogram displayed strong predictive performance for HBV-ALF development. Gene set enrichment analysis indicated that these genes were involved in retinol metabolism and peroxisome signaling pathways, and were significantly associated with immune cell types including M1 macrophages, plasma cells, and neutrophils. These findings provide novel insights into the molecular mechanisms of HBV-ALF and highlight potential targets for future diagnostic and therapeutic strategies.

**Supplementary Information:**

The online version contains supplementary material available at 10.1038/s41598-025-19813-x.

## Introduction

Acute liver failure (ALF) refers to a clinical syndrome characterized by rapid hepatocyte injury leading to liver failure, manifested by coagulopathy and varying degree of mental status alteration, in the absence of pre-existing cirrhosis. The Hepatitis B virus (HBV) is a double-stranded DNA virus that is transmitted percutaneously, sexually, and perinatally. The outcome of HBV infection is influenced by host immunologic factors and viral characteristics^1^. Acute exacerbations of Hepatitis B, particularly during the immune clearance/active phase, can potentially precipitate ALF^2^. The prognosis of ALF due to Hepatitis B is poor, and in severe cases, liver transplantation may be required. Consequently, treatment with nucleotide analogues should be initiated immediately^3^. Given the unfavorable prognosis of HBV-related ALF and the limited clinical interventions available beyond liver transplantation. exploring novel key genes and mechanisms is critical to understanding the disease’s pathogenesis and molecular mechanisms. This exploration could provide valuable targets for clinical diagnosis and treatment.

NOD-like receptors (NLRs) are crucial pattern recognition receptors (PRRs) involved in the pathogenesis and progression of various hepatic disorders. The NOD1 receptor, an important member of the NLRs family, activates immune cells by detecting diaminopimelic acid (DAP), a degradation product of cell wall peptides from pathogenic or commensal bacteria^4^. NOD1 expression is widespread, detected not only in immune cells but also in various tissue cells, including epithelial, stromal, and endothelial cells. In the liver, NOD1 is expressed not only in immune cells but also in approximately 70% of hepatocytes, the predominant cell type in the liver, where it plays a critical role in hepatic immune defense^5^. Therefore, NOD1 activity is closely linked to the immune status of the liver.

The endoplasmic reticulum (ER) is one of the largest subcellular compartments, playing a key role in various biological functions, including protein folding during cytoplasmic translation, Ca^2+^ homeostasis, and the biosynthesis of steroids and lipids^6^. The folding capacity of the ER varies significantly across cell types. Cells with high secretion capacities have an extensively developed ER, enabling efficient protein folding. When the folding capacity of ER proteins is overwhelmed, it leads to ER stress (ERS)^7^. ERS triggers inflammatory responses and is generally considered a damage-associated molecular pattern (DAMP) due to its involvement in PRR-related immune reactions. Several studies have shown that NOD1 can sense DAMP-induced ERS and initiate immune gene expression; however, the exact mechanism through which NOD1 mediates ERS sensing remains unclear^8–10^. Under ERS conditions, secretory proteins accumulate in misfolded and aberrantly modified forms within the organelle, and when ERS exceeds the body’s control capacity, apoptosis occurs. Hepatocytes, which have a highly developed ER, are capable of secreting large quantities of proteins efficiently^11^. However, viral infections impose significant strain on the ER, leading to a higher prevalence of ERS in chronic hepatitis. ERS plays a pivotal role in regulating inflammatory responses and cellular apoptosis, and severe ERS can contribute to the onset and progression of ALF^12^. The precise molecular mechanism of ERS in HBV-ALF remain poorly understood, highlighting the need for further investigation.

It is essential to acknowledge that a complex and bidirectional interaction exists among HBV infection, ERS, and the NOD1 signaling pathway. Increasing evidence indicates that HBV infection profoundly disrupts protein homeostasis in host cells, particularly by interfering with the ubiquitination process, thereby initiating and amplifying ERS^13^. In turn, ERS has been shown to exert regulatory influences on the HBV life cycle through modulation of viral replication and expression of viral proteins, potentially contributing to the progression of HBV-associated inflammatory responses and carcinogenic processes^14,15^. Furthermore, the interplay between HBV and the host’s innate immune system plays a crucial role in viral pathogenesis. Recent studies indicate that HBV may employ multiple mechanisms to suppress NOD1 activation as a strategy for immune evasion, which could impair the host’s ability to mount an effective antiviral response and influence disease outcomes^16^. Therefore, a comprehensive investigation into the specific involvement of NOD1 in HBV-induced ERS is of critical importance for elucidating the underlying pathogenic mechanisms of HBV-ALF.

The study utilized transcriptome data from patients with HBV-ALF and normal controls available in the Gene Expression Omnibus (GEO) database. A series of bioinformatics approaches, including differential gene expression analysis and protein-protein interaction network construction, were employed to identify the key NOD1 gene and its associated ERS-related genes (ERGs) in HBV-ALF. Comprehensive investigations were then conducted to explore the functional enrichment and regulatory networks of these key genes, aiming to elucidating their potential roles and molecular mechanisms in the pathogenesis of HBV-ALF.

## Materials and methods

### Data source

In this study, HBV-ALF related datasets were obtained from the GEO database (https://www.ncbi.nlm.nih.gov/gds). The GSE38941 dataset included Liver tissue samples from 17 patients with HBV-ALF and 10 normal controls (not infected with HBV), while GSE62029 contained Liver tissue samples from 13 patients with HBV-ALF and 10 normal controls (not infected with HBV). Additionally, 347 ERGs were retrieved from GOBP “Regulation of Response to Endoplasmic Reticulum Stress” and GOBP “Response to Endoplasmic Reticulum Stress” categories in the MSigDB database, with 265 unique ERGs remaining after removing duplicates^17^.

### Acquistion and functional enrichment analysis of candidate genes

Differentially expressed genes 1 (DEGs1) were analyzed using the limma package (v 3.54.0) (adj.*p*value < 0.05 & | log2FC | > 0.5)^18^. DEGs1 were identified between HBV-ALF and control samples in GSE38941, and DEGs2 were identified between HBV-ALF and control samples in GSE62029. The up- and down-regulated genes in DEGs1 were intersected with the corresponding up- and down-regulated genes in DEGs2, resulting in a set of intersection genes. These intersection genes were then further intersected with the ERGs to identify key genes. To investigate the expression of NOD1 in GSE38941, its expression in HBV-ALF and control samples was explored using the ggplot2 package (v 3.4.4)^19^. The correlation between key intersection genes and NOD1 was analyzed using Spearman correlation, with genes satisfying | R |>0.4 and *p* < 0.05 considered as candidate genes. The biological functions of the candidate genes were further investigated through Gene Ontology (GO) and Kyoto Encyclopedia of Genes and Genomes (KEGG) enrichment analyses using the clusterProfiler package (v 4.7.1.003)^20^. To further prioritize the candidate genes, we constructed a protein-protein interaction (PPI) network using the STRING database (https://string-db.org/) with a minimum interaction score threshold of 0.7. The resulting network was imported into Cytoscape software (v 3.9.1)^21^ for visualization and analysis. We then applied the Bottleneck algorithm from the cytoHubba plugin to rank the nodes (genes) based on their topological importance within the network. The Bottleneck algorithm identifies key nodes that act as bridges in the network, which are critical for maintaining network connectivity. The analysis was performed using the default parameters of cytoHubba, which calculates the bottleneck score for each node without requiring additional user-defined settings. The top 20 genes with the highest bottleneck scores were selected as candidate key genes for subsequent machine learning analysis.

### Identification and evaluation of key genes

Boruta analysis was performed using the Boruta package (v 8.0.0) to identify feature genes^22^ followed by Random Forest (RF) analysis using the randomForest package (v 4.7–1.1)^23^ to further screen key genes. To assess the ability of the key genes to distinguish between HBV-ALF and control samples, ROC curves were generated using GSE38941 and GSE62029 datasets. Finally, the expression of key genes in HBV-ALF patients and control samples from both datasets was analyzed.

### Construction of a nomogram model

To evaluate the diagnostic potential of the key genes for HBV-ALF, a nomogram was constructed based on their expression in GSE38941. The diagnostic performance of the nomogram was assessed using ROC curves.

### Functional analysis of key genes

The GeneMANIA database was utilized to predict genes associated with the function of the key genes and the involved biological functions. Additionally, differential analysis was performed based on the high and low expression groups of the key genes in GSE38941, with log2FC values ranked from largest to smallest. Gene Set Enrichment Analysis (GSEA) was then conducted using the clusterProfiler package (v 4.7.1.003) (*p* < 0.05)^20^ with the c2.cp.kegg.v7.4.symbols.gmt gene set from MSigDB as the reference.

### Immune infiltration analysis

To explore immune cells infiltration in GSE38941, the CIBERSORT algorithm was employed to calculate the relative abundance of 22 immune cell types in all samples. Differences in immune cell infiltration were assessed, with immune cells expressing 0 in 75% of the samples being excluded. Subsequently, the correlation between the remaining immune cells and key genes was explored, as well as the relationships between different immune cells.

### Inflammatory factor analysis and tissue localisation analysis

In this study, 43 inflammatory factors were retrieved from references, and 65 factors were derived from family members of FGFBasic and Eotaxin to explore their associations with key genes. The expression differences of 38 inflammatory factors between HBV-ALF and control samples in GSE38941 were examined using Wilcoxon’s method. Furthermore, the correlation between these inflammatory factors and the key genes was analyzed using Spearman’s correlation. The BIOGPS database was used to explore the role of key genes in various tissue and organ with a correlation cutoff of 0.9.

### Construction of regulatory network

To elucidate the molecular regulatory mechanisms of key genes, miRNAs targeting the key genes were predicted using the miRDB and Targetscan database within the multiMiR package (v 1.20.0)^24^. The predicted miRNAs from both databases were intersected to identify target miRNAs. Subsequently, the lncRNAs associated with these miRNAs were predicted using the Starbase database. An lncRNA-miRNA-key gene regulatory network was constructed using Cytoscape software. Additionally, potential drugs and Chinese medicines interacting with the key genes were predicted using the DGIdb and Coremine Medical database, respectively. To further explore the relationship between the key genes and other diseases, the CTD database was utilized to identify diseases associated with the key genes.

### Statistical analysis

All bioinformatics analyses were performed using R software (v 4.1.0), with differences between groups assessed by Wilcoxon’s method (*p* < 0.05).

## Results

### A total 20 candidate key genes were screened

**Fig. 1:**
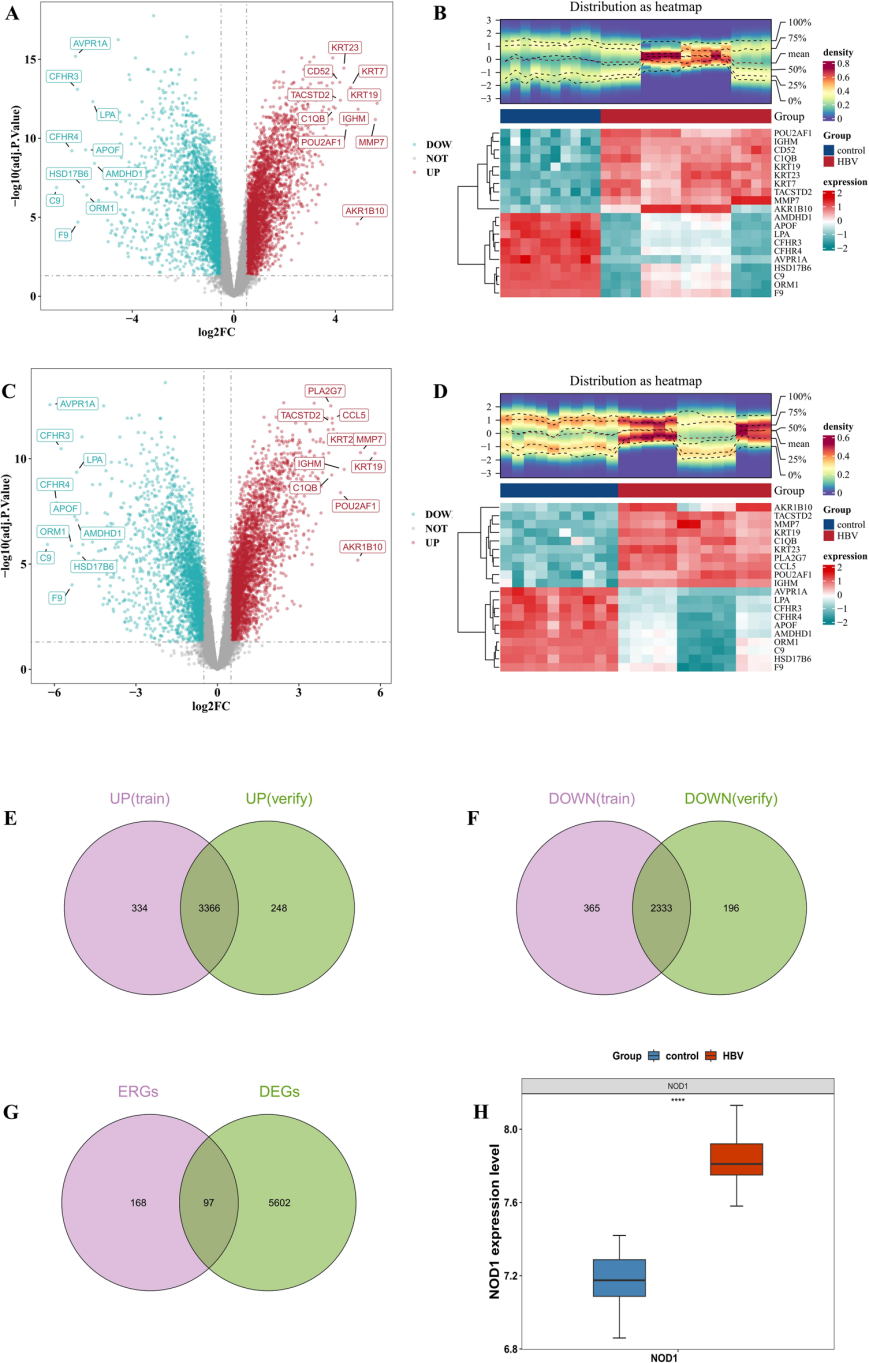
Acquisition of candidate genes. (**A**-**D**) Volcano plots and expression heatmaps of differential genes. (**E**-**G**) Venn diagrams showing the overlap of candidate genes. (**H**) Expression levels of NOD1 in patients with HBV-ALF and healthy controls.

After differential expression analysis, 6,398 DEGs1 in GSE38941 (Fig. 1A and B) and 6,143 DEGs2 from GSE62029 (Fig. 1C and D) were identified. Of these, 3,700 up-regulated and 2,698 down-regulated genes in DEGs1 were intersected with 3,614 up-regulated and 2,529 down-regulated genes in DEGs2 (Fig. 1E and F), resulting in 5,699 intersection genes. These intersection genes were then overlapped with 265 ERGs to identify 97 key genes (Fig. 1G). Additionally, the expression level of NOD1 was significantly higher in the HBV-ALF group compared to the control (Fig. 1H). Correlation analysis revealed a total of 89 candidate genes (Table 1).

**Table 1:** Correlation analysis of NOD1 and DE-ERGs.

Gene	*R*	*P* value	*P* value-sig
ABCA7	0.78472117	1.26E-06	***
ABL1	0.670485824	0.000129789	***
AIFM1	−0.900381981	1.60E-10	***
ALOX5	0.870933343	3.46E-09	***
AMFR	−0.873968877	2.62E-09	***
ANKS4B	−0.857033846	1.15E-08	***
APAF1	0.429335376	0.025428085	*
AQP11	−0.884815196	9.03E-10	***
ATP2A2	−0.70027501	4.77E-05	***
ATP2A3	0.728981997	1.61E-05	***
BAK1	0.769712714	2.69E-06	***
BAX	0.764553103	3.44E-06	***
BCAP31	0.514359938	0.006053822	**
BCL2	0.816223686	2.10E-07	***
BOK	−0.763869793	3.55E-06	***
CALR	−0.680311665	9.45E-05	***
CAV1	0.828113073	9.75E-08	***
CCDC47	−0.881741798	1.23E-09	***
CFTR	0.889126617	5.74E-10	***
CLGN	−0.810142135	3.05E-07	***
CLU	−0.812586003	2.63E-07	***
CREB3L3	−0.768138159	2.90E-06	***
CTH	−0.815210903	2.24E-07	***
CXCL8	0.624923757	0.000492396	***
DDIT3	−0.409813532	0.033758443	*
DERL3	0.833129584	6.93E-08	***
DNAJB9	−0.789248773	9.95E-07	***
DNAJC3	−0.774366061	2.14E-06	***
EDEM1	−0.763708649	3.57E-06	***
EIF2AK3	−0.59969443	0.000945927	***
ERLIN1	−0.890959238	4.71E-10	***
ERLIN2	−0.623396573	0.000513061	***
ERP44	−0.752788397	5.89E-06	***
FAM8A1	−0.756377046	5.01E-06	***
FBXO17	−0.884069132	9.75E-10	***
FCGR2B	0.878723172	1.66E-09	***
GORASP2	−0.74682965	7.66E-06	***
HSPA5	−0.910313227	4.56E-11	***
HYOU1	−0.833486137	6.76E-08	***
LPCAT3	−0.899648789	1.75E-10	***
MAN1A1	−0.723995799	1.97E-05	***
MAN1A2	−0.566366334	0.002071918	**
MAN1C1	0.849725265	2.06E-08	***
MANF	−0.735828886	1.22E-05	***
MARCKS	0.830456785	8.32E-08	***
MBTPS2	−0.68892285	7.08E-05	***
NCK2	0.844227402	3.12E-08	***
NGLY1	−0.714111312	2.87E-05	***
NOD1	1	3.38E-197	***
NOD2	0.788911034	1.01E-06	***
NR1H3	0.740644648	9.98E-06	***
NRBF2	−0.840995964	3.96E-08	***
NUPR1	−0.561936824	0.002285514	**
P4HB	−0.779780231	1.63E-06	***
PARP16	−0.705010726	4.02E-05	***
PDIA3	−0.812891486	2.58E-07	***
PDIA4	−0.832416782	7.28E-08	***
PDIA6	−0.779254543	1.67E-06	***
PIK3R1	−0.522383505	0.005186425	**
PMAIP1	0.803116015	4.61E-07	***
PML	0.747784942	7.35E-06	***
PPP1R15B	−0.843721396	3.24E-08	***
PPP2CB	−0.650061125	0.000242366	***
PTPN2	0.809102167	3.24E-07	***
RCN3	0.857950296	1.07E-08	***
RNF103	−0.695034385	5.74E-05	***
RNF185	−0.7425864	9.19E-06	***
RNFT1	−0.692730734	6.21E-05	***
RNFT2	0.769747908	2.68E-06	***
SDF2L1	−0.65455113	0.000212108	***
SEC16A	−0.818848413	1.78E-07	***
SEC61B	−0.816903571	2.01E-07	***
SEL1L	−0.639266623	0.000331115	***
SELENOK	−0.447754375	0.019179592	*
SESN2	−0.658312958	0.00018937	***
SRPX	0.684789376	8.14E-05	***
THBS1	0.547342802	0.003128329	**
TMCO1	−0.756988013	4.88E-06	***
TMEM33	−0.822639818	1.40E-07	***
TMTC3	−0.482584807	0.010788797	*
TMTC4	−0.494576018	0.008729632	**
TMUB2	0.702826593	4.35E-05	***
TNFRSF10B	−0.518110964	0.00563418	**
TRIB3	−0.854001381	1.47E-08	***
UBA5	−0.832543936	7.22E-08	***
UBQLN1	−0.867532478	4.70E-09	***
UBXN1	0.826153415	1.11E-07	***
UFL1	−0.678887902	9.90E-05	***
UFM1	−0.49358592	0.008886121	**

* represents *p* < 0.05, ** represents *p* < 0.01, *** represents *p* < 0.001.

**Fig. 2:**
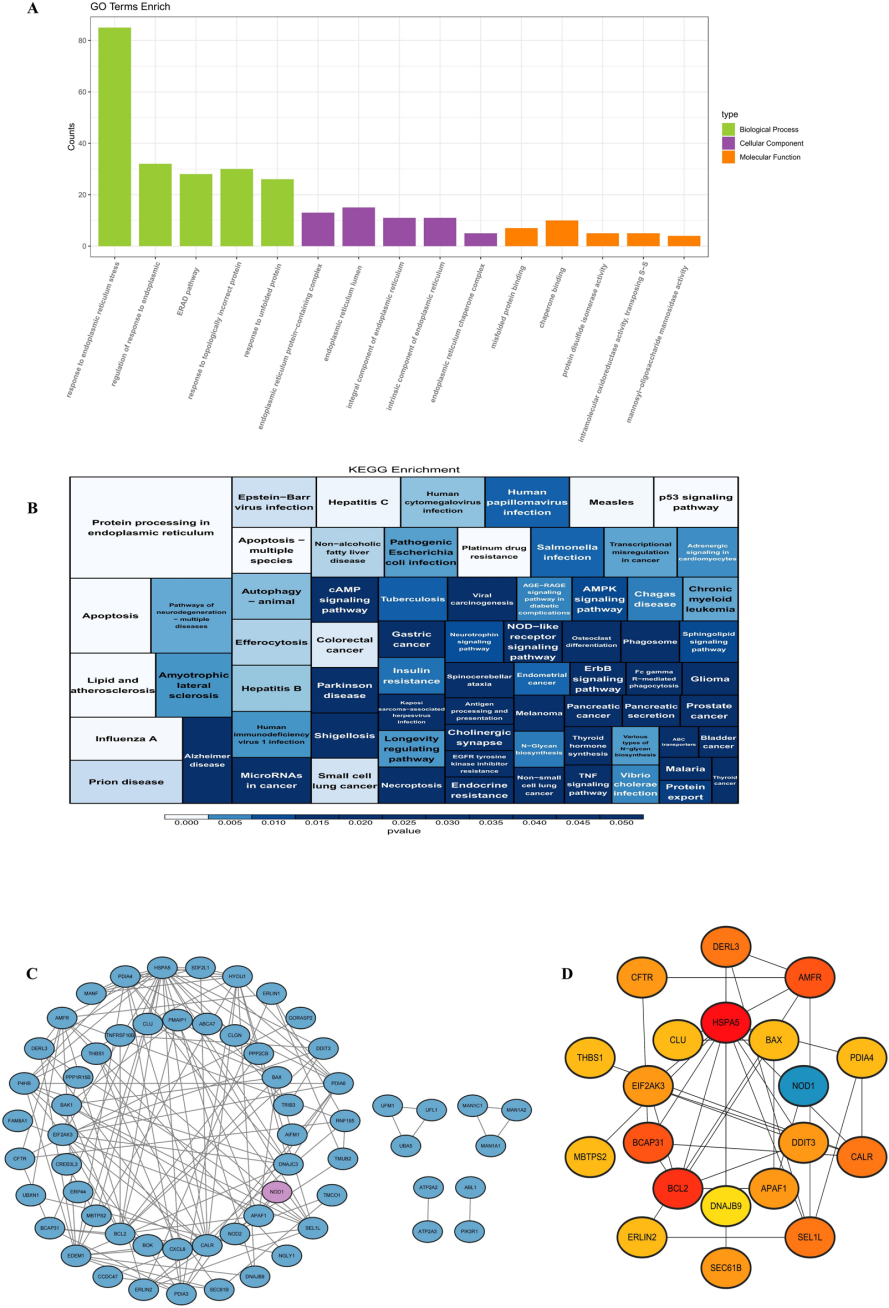
Functional enrichment analysis of candidate genes. (**A**) Gene Ontology (GO) enrichment analysis of candidate genes. (**B**) Kyoto Encyclopedia of Genes and Genomes (KEGG) pathway analysis of candidate genes. (**C**) Protein-protein interaction (PPI) network of candidate genes. (**D**) Top 20 genes scored by the BottleNeck algorithm.

The candidate genes were enriched in 735 functional entries based on the GO database (BP: 644, CC: 38, MF: 53), including “response to endoplasmic reticulum stress” (BP), “endoplasmic reticulum protein-containing complex” (CC), and “misfolded protein binding” (MF) (Fig. 2A). In the KEGG database, these genes were involved in 71 signaling pathways, such as “protein processing in endoplasmic reticulum” and “apoptosis” (Fig. 2B). Furthermore, 61 candidate genes formed 131 interaction pairs in the PPI network, including ATP2A2-ATP2A3 and UBA5-UFL1 (Fig. 2C). The top 20 genes identified by the BottleNeck algorithm were considered as candidate key genes for subsequent analysis (Fig. 2D).

### The diagnostic performance of key genes was wonderful

**Fig. 3:**
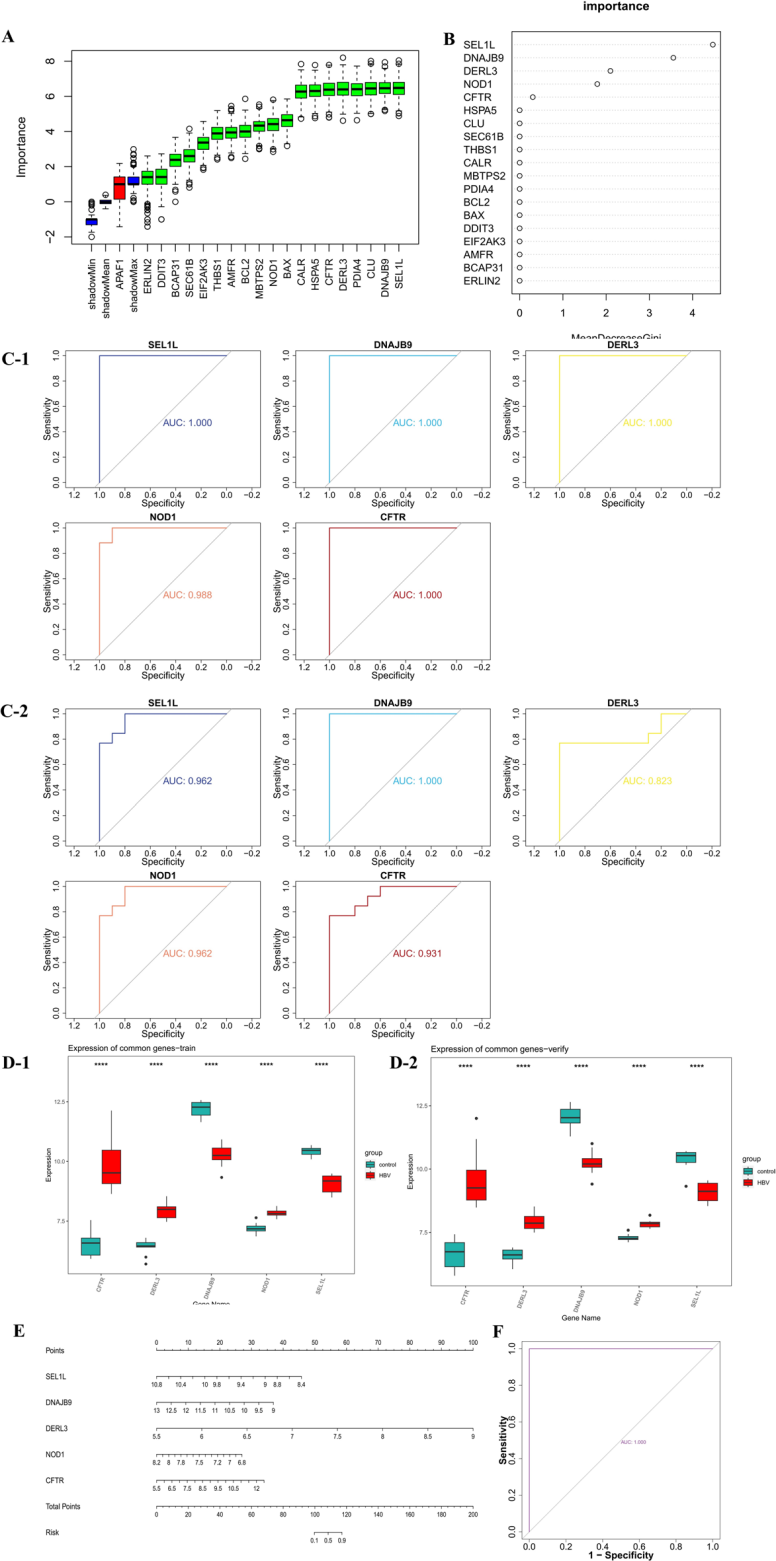
Acquisition and diagnostic performance of key genes. (**A**-**B**) Identification of 5 key genes through Boruta analysis and Random Forest. (**C**) ROC curve analysis of key genes in the training set (GSE38941) and validation set (GSE62029). (**D**) Expression levels of key genes in the training set (GSE38941) and validation set (GSE62029). (**E**) Nomogram for key genes. (**F**) ROC curve of the nomogram.

In the Boruta analysis, 19 feature genes (e.g., HSPA5, DERL3, SEL1L, NOD1) were selected for the RF model (Fig. 3A), with the top 5 genes—SEL1L, DNAJB9, DERL3, NOD1, and CFTR—identified as key genes (Fig. 3B). ROC analysis showed AUC values exceeding 0.8 in both GSE38941 and GSE62029, indicating excellent diagnostic performance of the key genes in distinguishing HBV-ALF from control samples (Fig. 3C). Furthermore, in both datasets, CFTR, DERL3, and NOD1 exhibited significantly higher expression in the HBV-ALF group, while DNAJB9 and SEL1L were expressed at lower levels (Fig. 3D). Subsequently, a nomogram was constructed to predict HBV-ALF occurrence (Fig. 3E), and the AUC value for the nomogram exceeded 0.9, further confirming its strong diagnostic performance (Fig. 3F).

### Key genes played important roles in retinol metabolism and peroxisome

**Fig. 4:**
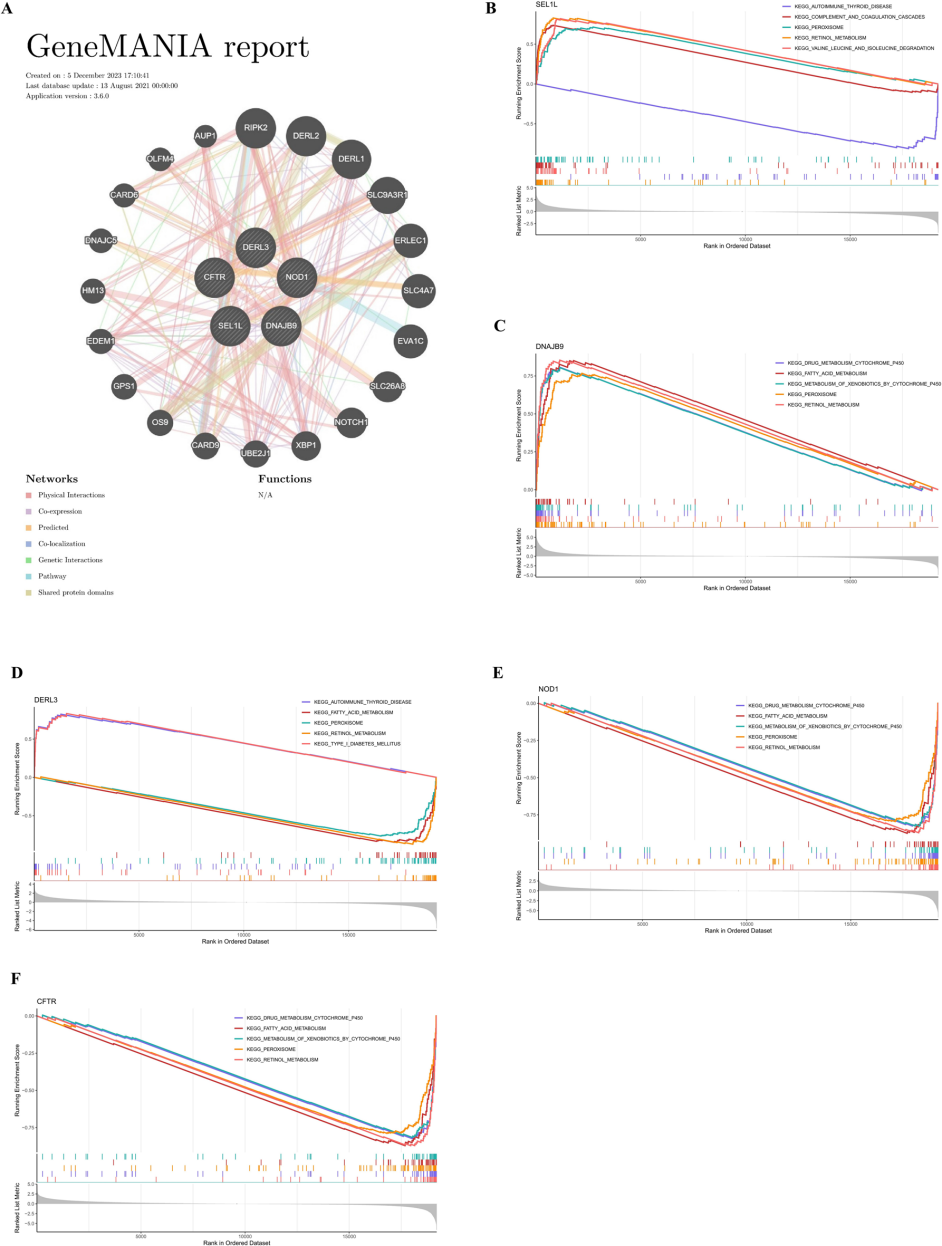
Functional analysis of key genes. (**A**) GeneMANIA network diagram. (**B**-**F**) Gene Set Enrichment Analysis (GSEA) for each key gene.

Using the GeneMANIA database, a total of 20 genes related to the functions of the key genes were predicted, all of which were involved in 37 biological functions, including ER to cytoplasm transport and the ERAD pathway (Fig. 4A). GSEA analysis further revealed that all key genes were significantly involved in retinol metabolism and peroxisome signaling pathways (Fig. 4B and F).

### A total of 4 differential immune cells correlate with key genes

**Fig. 5:**
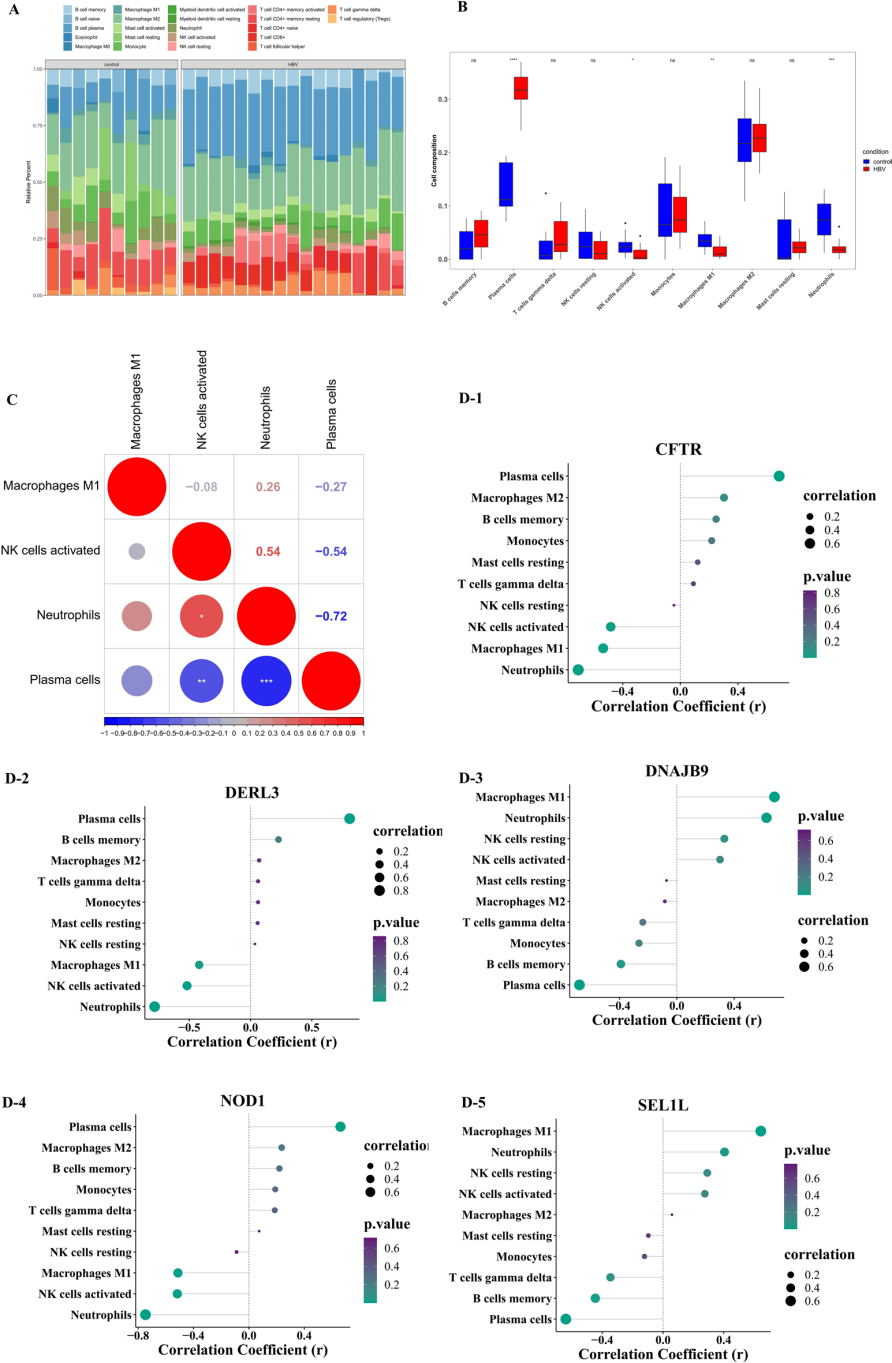
Immune infiltration analysis. (**A**) Immune cell infiltration accumulation diagram. (**B**) Boxplots showing immune cell differences between normal and HBV-ALF groups. (**C**) Correlation of differential immune cells. (**D**) Correlation between the 5 key genes and immune-infiltrating cells.

The infiltration abundance of 22 immune cell types was calculated (Fig. 5A), and after eliminating immune cells with zero expression in 75% of the samples, 10 immune cell types remained for further analysis. Among these, four immune cells—M1 macrophages, activated natural killer (NK) cells, neutrophils, and plasma cells—showed significantly different expression between the HBV-ALF and control groups (Fig. 5B). Correlation analysis revealed that neutrophils were significantly positively correlated with activated NK cells (cor = 0.54, *p* < 0.05) and negatively correlated with plasma cells (cor = −0.72, *p* < 0.05), while plasma cells and activated NK cells were also negatively correlated (cor = −0.54, *p* < 0.05) (Fig. 5C). Additionally, the key genes were significantly correlated with M1 macrophages, plasma cells, and neutrophils (|R| > 0.4, *p* < 0.05) (Fig. 5D).

### Key genes play a role in different tissues

**Fig. 6:**
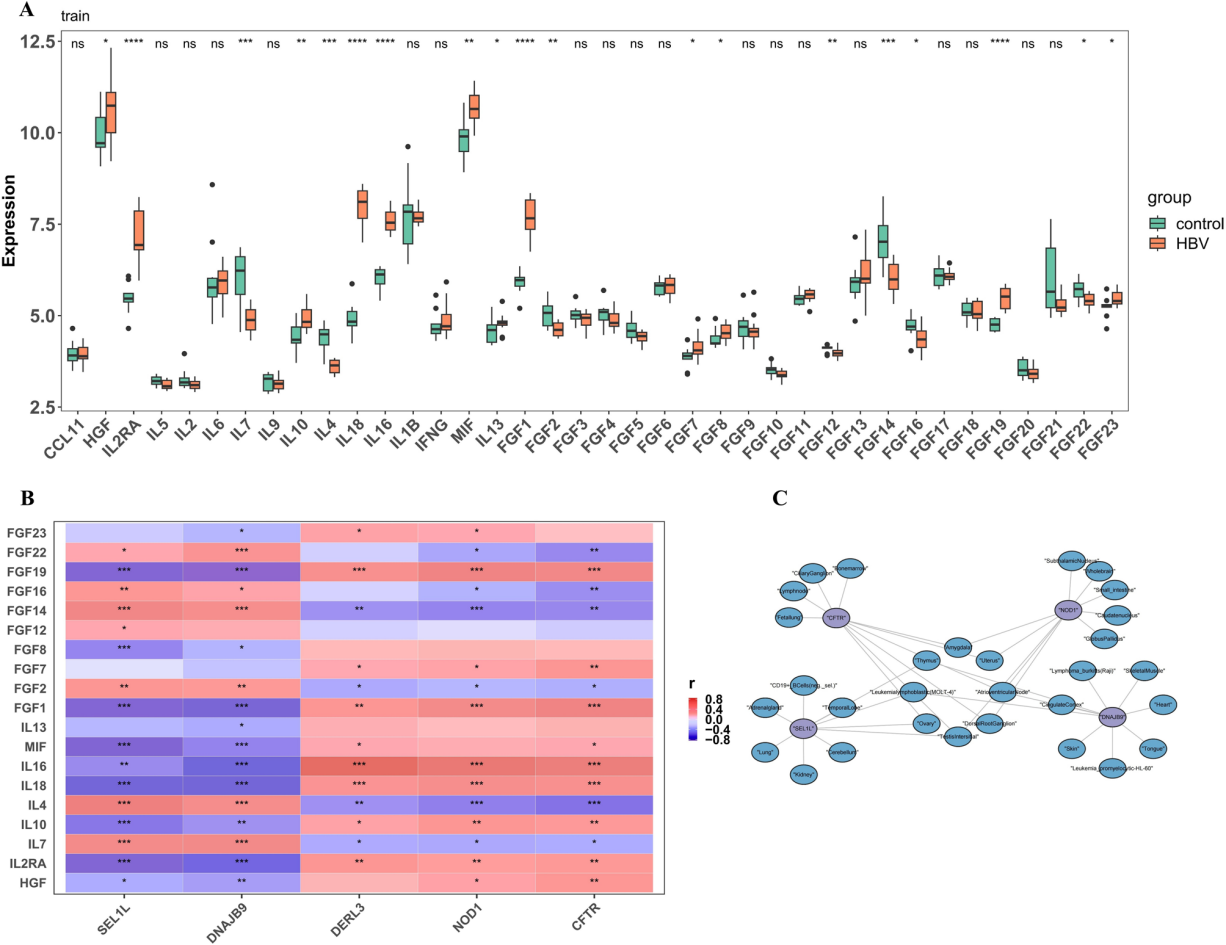
Inflammatory factor and tissue localization analysis. (**A**) Boxplot of inflammatory factor expression. (**B**) Heatmap showing the correlation between inflammatory factors and key genes. (**C**) Network diagram of key genes and their associated tissues/organs.

In the analysis of 38 inflammatory factors, 19 factors (e.g., HGF, IL2RA, IL7) exhibited significant expression differences between the HBV-ALF and control groups (Fig. 6A). Correlation analysis revealed positive correlations between DERL3, NOD1, CFTR, and IL16, DNAJB9 and IL7, as well as SEL1L and IL4. The most significant negative correlations were observed between NOD1, CFTR, and IL4, DERL3 and IL18, SEL1L and FGF1, and DNAJB9 and FGF19 (Fig. 6B). Tissue localization analysis indicated that key genes played important roles in various tissues, such as SEL1L in the thymus and DNAJB9 in the skin (Fig. 6C). As a central immune organ, the thymus contains SEL1L, which may affect the systemic immune imbalance in HBV-ALF by regulating the ER stress response of T cells. Meanwhile, DNAJB9 in the skin suggests that this chaperone may play a conserved role in the ER stress of keratinocytes.

### The lncRNA-miRNA-key gene, drug networks and disease networks of key genes were constructed

**Fig. 7:**
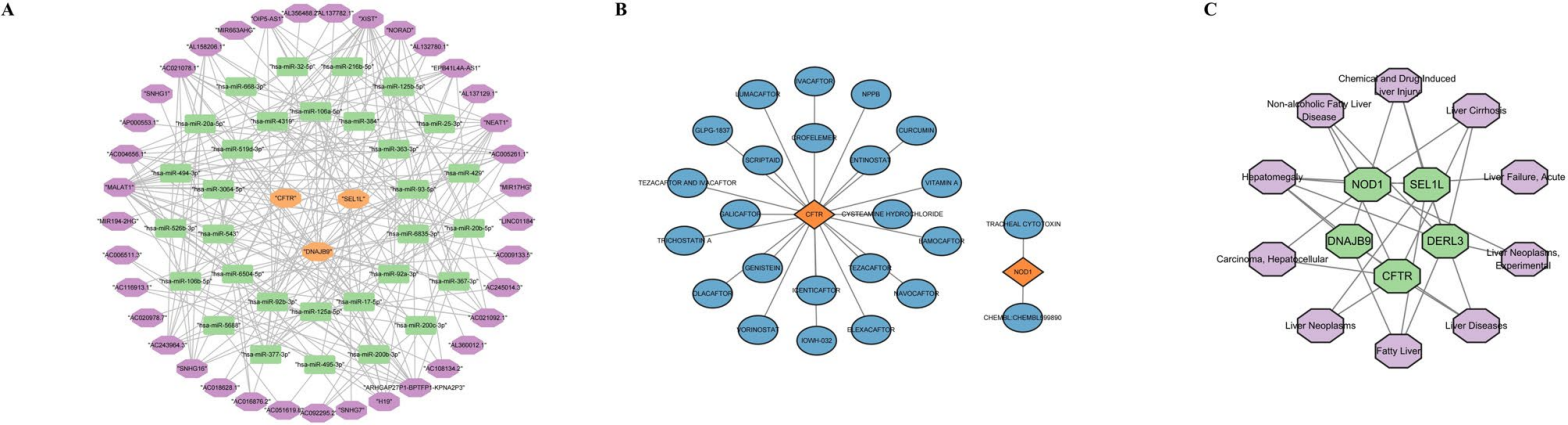
lncRNA-miRNA-key gene, drug networks, and disease networks of five key genes. (**A**) ceRNA regulatory network. **(B**) Drug prediction network. (**C**) Disease prediction network.

MiRNA analysis identified 143 miRNAs from miRDB, which were overlapped with 49 miRNAs from TargetScan, resulting in 32 target miRNAs. Based on these, 37 lncRNAs were predicted from the Starbase database. A lncRNA-miRNA-key gene regulatory network was constructed, including 3 key genes (SEL1L, DNAJB9, CFTR), 32 miRNAs, and 37 lncRNAs. Key relationships in this network included AC018628.1-hsa-miR-377-3p-DNAJB9 and MIR663AHG-hsa-miR-106a-5p-SEL1L (Fig. 7A). Drug predictions from the DGIdb revealed 25 drugs (e.g., Lumacaftor, Tezacaftor) corresponding to CFTR, and 2 drugs (CHEMBL599890, Tracheal Cytotoxin) corresponding to NOD1 (Fig. 7B). Additionally, Chinese medicine predictions identified 1 drug (Xi Wu Wei Zi) for CFTR, 1 drug (Hai Shen) for SEL1L, 4 drugs (e.g., Mai Dong, Teng Huang) for DNAJB9, and 4 drugs (e.g., Du Huo, Long Xian Xiang) for NOD1 (Table 2). Finally, disease associations with the key genes were predicted based on the CTD database, including chemical and drug-induced liver injury and hepatomegaly (Fig. 7C).

**Table 2:** Key genes and corresponding traditional Chinese medicine.

gene	drug	pvalue
CFTR	xi wu wei zi	0.0441
SEL1L	hai shen	0.0113
DNAJB9	mai dong	1.28E-05
DNAJB9	teng huang	0.00124
DNAJB9	mang guo he	0.00887
DNAJB9	jiang dou	0.00895
NOD1	du huo	0.00126
NOD1	long xian xiang	0.00925
NOD1	xun lu xiang	0.0132
NOD1	han niu cao	0.0132

## Discussion

Although ALF is a rare occurrence, its mortality rate can exceed 50%^25^. Currently, clinical treatment for this high-mortality disease primarily focuses on supportive therapy and the management of various complications. However, due to the disease’s rapid progression, many patients eventually require Liver transplantation. Less than 10% of hepatobiliary transplant recipients receive treatment specifically targeting ALF, as patients require extensive pre-transplant evaluation and preparation while their condition deteriorates rapidly^26,27^. The innate immune system is the primary defense mechanism against pathogen invasion and plays a pivotal role in acute inflammation triggered by microbial infection or tissue damage. Immune cells initiate immune responses by recognizing specific molecular structures expressed by pathogens, known as pathogen-associated molecular patterns (PAMPs), or by detecting fixed structures expressed by damaged tissues and necrotic cells, referred to as DAMPs, through PRRs. The NOD1 receptor is a key member of the PRR family, with extensive expression across various hepatic cell types. Our previous research has shown its involvement in the regulation of LPS-GalN-induced ALF^28^. Based on this, a close correlation may exist between NOD1, its associated genes, and the initiation of ALF.

Beyond its conventional role as a receptor for bacterial peptidoglycan fragments, previous studies have highlighted NOD1’s significant involvement in ERS mechanisms. Chemical agents such as thapsigargin and dithiothreitol induce an increase in IL-6 levels during ERS-induced inflammation, partly dependent on the presence of NOD1. Additionally, infection with Brucella abortus triggers an inflammatory response through ERS promotion, which can be mitigated by tauroursodeoxycholate, an ERS inhibitor. NOD1 plays a pivotal role in this process^8^.

In addition to NOD1, four key genes associated with it were identified in this study. Among these, SEL1L and DNAJB9 were expressed at significantly lower levels in the HBV-ALF group. SEL1L, located on the ER membrane, regulates the stability of the E3 ligase Hrd1. The SEL1L-Hrd1-mediated ER-associated degradation (ERAD) pathway is crucial in regulating fundamental physiological processes such as lipid metabolism, water homeostasis, food intake regulation, and systemic energy balance^29^. Furthermore, the SEL1L-related ERAD pathway is closely linked to the suppression of hepatocyte proliferation^30^. The DNAJ protein, a member of the heat shock protein 40 (HSP40) family of molecular chaperones, is essential in various cellular processes, including protein folding, unfolding, translation, degradation, and intracellular signaling functions^31^. DNAJB9, classified as a type II DNAJ homolog subfamily B member, functions as a negative feedback regulator, suppressing the proapoptotic activity of P53^32^. Among the 5 key genes, DERL3, NOD1, and CFTR were expressed at significantly higher levels in the HBV-ALF group. DERL3, a member of the Derlin family, is primarily localized to the ER membrane^33^. Previous studies have indicated that DERL3 plays a role in the metastasis and apoptosis of colorectal, gastric, and breast cancers. It has also been shown to be primarily involved in the ERAD process^33–36^.

The CFTR gene encodes an anion channel that is activated by phosphorylation and cAMP-dependent signaling, facilitating the transport of chloride and bicarbonate ions across the apical plasma membrane of epithelial cells. It is widely expressed in various tissues throughout the body^37^. Like most polytopic membrane proteins, CFTR biogenesis occurs in the ER and involves the coordinated folding of its individual domains within three cellular compartments: the ER membrane, the ER Lumen, and the cytosol. This compartmentalization begins as the nascent polypeptide emerges from the ribosome, followed by the assembly of transmembrane and nucleotide-binding domains into their final folded structure. This process takes approximately 30–120 min and is assisted by various cytosolic and luminal chaperones, including HSP70, HSP40, HSP90, and calnexin^38^. Mutations in CFTR cause cystic fibrosis, a prevalent autosomal recessive disease with life-threatening consequences, predominantly observed in Caucasian populations^39^. Studies have also reported that CFTR dysfunction is associated with the development and progression of esophageal cancers, including esophageal squamous cell carcinoma (ESCC) and esophageal adenocarcinoma (EAC), with a tumor-suppressive role for CFTR being supported across studies^40–42^. Additionally, CFTR, as a hypermethylated hub gene, exhibits low expression in hepatocellular carcinoma^43,44^. Furthermore, CFTR has been shown to regulate Src tyrosine kinase activity, governing biliary epithelial inflammation by inhibiting TLR4 phosphorylation and subsequent NF-κB activation^45,46^.

This study identified five key genes exhibiting distinct expression patterns in HBV-ALF: CFTR, DERL3, and NOD1 were significantly upregulated, whereas DNAJB9 and SEL1L were markedly downregulated. This differential expression profile suggests a complex interplay between ERS and immune-inflammatory responses during HBV infection. The upregulation of DERL3 reflects activation of the ERAD pathway, indicating that hepatocytes may attempt to alleviate ERS by enhancing the clearance of misfolded proteins^47^. Increased expression of NOD1 implies that ERS may serve as a DAMP, which can be recognized by the innate immune system and subsequently activate pro-inflammatory signaling cascades^9,48^. The upregulation of CFTR may represent a compensatory mechanism aimed at suppressing excessive inflammatory responses through modulation of signaling pathways such as SRC/NF-κB; however, its dysregulation or functional impairment could also contribute to disease pathogenesis^49^. Conversely, the downregulation of SEL1L and DNAJB9 likely has more profound pathological implications. As a core component of the ERAD complex (SEL1L) and a key endoplasmic reticulum molecular chaperone (DNAJB9), their reduced expression indicates a significant impairment in ER proteostasis^50,51^. Notably, diminished DNAJB9 levels may compromise its capacity to inhibit p53-mediated apoptosis, thereby promoting ERS-induced hepatocyte death^32^. Collectively, these bidirectional changes in gene expression may disrupt the homeostatic balance of the ER and hepatocyte function, potentially contributing to the exacerbation of inflammatory responses and the loss of control over apoptotic processes in HBV-ALF.

This study revealed that peroxisomal and retinol metabolism pathways were enriched with all key genes. Peroxisomes, multifunctional organelles present in nearly all eukaryotic cells, also express Mitochondrial antiviral signaling protein (MAVS), which enhances antiviral responses mediated by intracellular RLR family members^52^. Furthermore, the cytokine-induced inflammatory storm and subsequent pathological processes are major contributors to ALF. Previous studies have shown that during the early phase of acute liver injury, retinol metabolism is closely linked to the substantial release of pro-inflammatory cytokines, such as TNF-α, IL-1β, and IL-6^53^. These pathways may thus represent critical mechanisms through which the key genes contribute to the development and progression of HBV-ALF.

In immune cell infiltration analysis, a significant increase in plasma cell infiltration was observed in patients with HBV-ALF, while the abundance of activated NK cells, M1 macrophages, and neutrophils significantly decreased. As immune cell abundance varies across different stages of ALF, the early phase is marked by heightened pro-inflammatory activity to facilitate the clearance of harmful antigens. Therefore, the observed reduction in M1 macrophages and neutrophils may indicate advanced disease progression in a subset of patients, as opposed to reflecting a true protective downregulation. Upon migration into the liver parenchyma, neutrophils interact with hepatic cells, significantly increasing the generation and secretion of reactive oxygen species. This process induces localized hepatocyte damage and contributes to extensive liver necrosis^54^. Neutrophils also release pro-inflammatory cytokines, chemokines, and granule proteins that interact with other innate immune cells, including circulating mononuclear macrophages, Kupffer cells, and NK cells^55,56^. These interactions further intensify the sterile inflammatory response, exacerbating liver tissue damage. As the inflammatory storm progresses and inflammatory factors damage normal liver cells, the liver enters the immune tolerance stage. During this phase, immune cells are rapidly consumed or migrate to other tissues and organs, contributing to multi-organ damage^57^. As ALF progresses rapidly, patients often enter the immune tolerance and systemic inflammatory response stages by the time they undergo diagnostic evaluations. Consequently, in this study, most pro-inflammatory cells showed decreased levels in patients with ALF. In the correlation analysis between key genes and immune cells, all five key genes demonstrated significant correlations with M1 macrophages, plasma cells, and neutrophils. Additionally, NOD1, DERL3, and CFTR showed significant correlations with activated NK cells. Considering the critical role of these immune cells in ALF pathogenesis^57,58^ these findings suggest that the key genes play a pivotal role in regulating immune responses during ALF. However, it is important to emphasize that the current analysis of immune cell abundance reflects relative levels across the entire tissue. As non-immune cells are actively involved in the pathophysiological process of ALF, the observed immune signatures may be modulated by activated, stressed, or dying non-immune cells, which could introduce potential confounding factors in the interpretation of immune gene expression profiles. Therefore, future investigations integrating spatial transcriptomics or multi-omics methodologies to precisely delineate the spatial organization and interactions between immune and non-immune cellular components, will constitute a crucial avenue for advancing our understanding of the disease.

In the analysis of potential drug predictions for key genes in HBV-ALF, CFTR was the only gene for which relatively well-established drugs were identified: Lumacaftor, Tezacaftor, and Crofelemer. No well-defined potential drugs were predicted for the other genes. Lumacaftor and Tezacaftor are CFTR correctors targeting the F508del-CFTR mutation, used in combination with Ivacaftor to address the most common CFTR mutation, F508del^59,60^. While their mechanisms of action are not fully understood, several studies suggest they may help repair the abnormal assembly of the full-length protein, enhancing its processing, trafficking, and stability^61^. Crofelemer, a botanical antidiarrheal agent, is used to treat noninfectious diarrhea in HIV-positive patients receiving antiretroviral medications. In preregistration studies, serum transaminase elevations were observed in 2.7% of treated subjects^62^. Despite the lack of literature confirming the role of these drugs in ALF, several studies have explored the use of lumacaftor/ivacaftor in the treatment of cystic fibrosis-related liver disease. In randomized controlled clinical trials, some patients experienced elevated serum alanine aminotransferase and aspartate aminotransferase levels following treatment; however, these biomarkers generally returned to baseline after discontinuation of therapy. Moreover, emerging evidence suggests that lumacaftor/ivacaftor may improve liver steatosis. Nevertheless, the European Medicines Agency has reported post-marketing cases of severe adverse outcomes, including liver failure and death, in cystic fibrosis patients with pre-existing cirrhosis and portal hypertension who were treated with lumacaftor/ivacaftor. Overall, clinical evidence supporting the use of these drugs in liver diseases remains limited. That said, existing evidence suggests that these agents may modulate hepatic immune or metabolic pathways. Therefore, further research is warranted to systematically investigate their mechanisms of action and safety profiles across diverse liver disease contexts^62–64^. To identify potential Chinese patent medicines related to key genes for HBV-ALF treatment, several traditional Chinese medicine ingredients were predicted, including xi wu wei zi, hai shen, mai dong, teng huang, mang guo he, jiang dou, du huo, long xian xiang, xun lu xiang, and han niu cao. However, due to the complexity of traditional Chinese medicine compositions and their varied modes of administration, the specific effects and mechanisms of action of these ingredients in ALF remain unclear and warrant further investigation.

However, this study has several limitations that merit careful consideration. First, the analysis was conducted using publicly available transcriptomic datasets, which lack comprehensive clinical metadata—such as age, sex, viral genotype, and disease stage—potentially limiting the generalizability and external validity of the findings. Furthermore, the control group consisted of individuals without HBV infection, which may confound the interpretation of HBV-specific gene expression changes. Second, the mechanisms of action of the candidate drugs identified through bioinformatics approaches remain to be experimentally validated within the specific pathophysiological context of HBV-ALF. Third, immune infiltration profiling and the construction of molecular regulatory networks were based on bulk RNA-seq data, which may obscure the dynamic heterogeneity of immune cell populations across disease stages and fail to capture stage-specific regulatory interactions. Additionally, immune infiltration analyses may be influenced by the presence of non-immune inflammatory cells, potentially introducing false-positive signals into the interpretation of immune signatures. Lastly, the expression patterns of key genes across four distinct immune cell types and clinical samples remain insufficiently characterized. To address these limitations, future research should prioritize the collection of well-annotated clinical samples with comprehensive demographic and clinical data, including HBV carriers without ALF as more appropriate controls, to better delineate HBV-specific effects. Second, the pharmacological effects and molecular mechanisms of the predicted drugs should be rigorously validated using in vitro cell models and in vivo experimental systems. Third, longitudinal immune monitoring at multiple time points or the application of single-cell transcriptomic technologies can enable the reconstruction of temporal immune dynamics and stage-specific regulatory networks, thereby minimizing interference from non-immune components. Finally, the expression profiles of key genes should be further validated using qPCR and immunohistochemistry in purified immune cell subsets and expanded clinical cohorts to precisely define their cellular localization and clinical correlations.

## Conclusion

The study elucidated the molecular mechanisms underlying HBV-ALF, with a particular focus on the regulatory role of the NOD1 receptor and its associated genes. Key findings include significant expression differences in patients with HBV-ALF, where SEL1L and DNAJB9 were notably downregulated, while DERL3, NOD1, and CFTR were upregulated. These genes contribute to disease progression through pathways such as ERS, ERAD, and immune regulation. Dynamic changes in immune cell infiltration were observed, with increased plasma cell infiltration and decreased levels of activated NK cells, M1 macrophages, and neutrophils. This suggests a potential shift in immune status from pro-inflammatory to immune tolerance as the disease progresses. Drug predictions indicated that CFTR is the only gene with established targeted therapies (e.g., Lumacaftor and Tezacaftor), while no mature therapies exist for other key genes. Additionally, certain herbal components may hold therapeutic potential, but their mechanisms need further validation. Future studies should incorporate experimental models (e.g., cellular or animal studies) to confirm gene functions, expand clinical sample sizes for improved result reliability, and explore multi-pathway synergy mechanisms to facilitate the clinical translation of potential therapeutic targets.

## Supplementary Information

Below is the link to the electronic supplementary material.

Supplementary Material 1

## Data Availability

All data generated or analyzed during this study were retrieved from the GEO database (https://www.ncbi.nlm.nih.gov/gds).

## Abbreviations

ERS: Endoplasmic reticulum stress
ALF: Acute liver failure
HBV: Hepatitis B virus
HBV-ALF: Hepatitis B virus-associated ALF
ERGs: ERS related genes
GSEA: Gene set enrichment analysis
PAMPs: Pathogen-associated molecular patterns
DAMPs: Damage-associated molecular patterns
PRRs: Pattern recognition receptors
NLRs: NOD-like receptors
DEGs1: Differentially expressed genes 1
RF: Random Forest
ERAD: ER-associated degradation
ESCC: Esophageal squamous cell carcinoma
EAC: Esophageal adenocarcinoma
MAVS: Mitochondrial antiviral signaling protein
