# Supplementary material for: Bioinformatics identification of key genes correlating NOD1 and Endoplasmic Reticulum stress in Hepatitis B virus-induced acute liver failure
Source: Sci Rep. 2025 Oct 14;15:35919. doi: 10.1038/s41598-025-19813-x (PMC12521598; doi:10.1038/s41598-025-19813-x)
Supplement: Supplementary file 1 — Supplementary Material 1 [file 41598_2025_19813_MOESM1_ESM.docx]

#### Supplementary materials

#### Key Genes Associated with NOD1-Mediated Endoplasmic Reticulum Stress in Hepatitis B-Induced Acute Liver Failure

Fuexue Deng^1^†, Wei Jiang^1^†, Ning Wang^2^, Yuchao Wu^2^, Jing Xu^2^, Rongrong Hou^2^*, Fang Jia^2^*

**Search parameters utilizing the GeneMANIA database (Figure 4A)**

**Organism** Homo sapiens (human)

**Genes** CFTR , SEL1L , DERL3 , NOD1 , DNAJB9 **Network** Automatically selected weighting method

**weighting**

**Networks A**

| Abbasi-Schild-Poulter-2019 , Abu-Odeh-Aqeilan-2014 , Achuthankutty-Mailand-  2019 , Agrawal-Sedivy-2010 , Ahn-Lee-2008 , Albers-Koegl-2005 , Alexander-Wang- 2018 , Alexandru-Deshaies-2008 , Alizadeh-Staudt-2000 , Alsulami-Cagney-2019 ,  An-Sun-2017 , Andresen-Flores-Morales-2014 , Arbogast-Gros-2019 , Arijs-  Rutgeerts-2009 , Arroyo-Aloy-2014 , Arroyo-Aloy-2015 , Asadi-Dhanvantari-2018  **B** |
| --- |
| Bailey-Hieter-2015 , Bandyopadhyay-Ideker-2010 , Banks-Washburn-2016 ,  Bantscheff-Drewes-2011 , Barr-Knapp-2009 , Barreiro-Alonso-Cerdán-2018 , Barrios-  Rodiles-Wrana-2005 , Behrends-Harper-2010 , Behzadnia-Lührmann-2007 ,  Benleulmi-Chaachoua-Jockers-2016 A , Benleulmi-Chaachoua-Jockers-2016 B , Bennett-Harper-2010 , Benzinger-Hermeking-2005 , Berggård-James-2006 , Bett- Hay-2013 , Beyer-Boldt-2018 , Bhatnagar-Attie-2014 , Bild-Nevins-2006 B ,  BIOGRID-SMALL-SCALE-STUDIES , BIOGRID-SMALL-SCALE-STUDIES , Bishof-Seyfried-2018 , Blandin-Richard-2013 , Blomen-Brummelkamp-2015 ,  Blomen-Brummelkamp-2015 , Bogachek-Weigel-2014 , Boldrick-Relman-2002 ,  Boldt-Roepman-2016 , Botham-Schimmer-2019 , Bouwmeester-Superti-Furga-2004 , Brady-Omary-2018 , Brajenovic-Drewes-2004 , Brehme-Superti-Furga-2009 ,  Burington-Shaughnessy-2008 , Butland-Hayden-2014 , Byron-Humphries-2012  **C** |
| Cai-Conaway-2007 , Camargo-Brandon-2007 , Campos-Reinberg-2015 , Cao-  Chinnaiyan-2014 , Carmon-Liu-2014 , Caron-van Attikum-2019 , CELL MAP ,  Chen-Brown-2002 , Chen-Ge-2013 A , Chen-Ge-2013 B , Chen-Guan-2018 , Chen- Huang-2014 , Chen-Krogan-2018 , Chen-Yu-2018 , Chen-Zhang-2013 , Chen-Zhou- 2019 , Cheng-DeCaprio-2017 , Chi-Reed-2018 , Chitale-Richly-2017 , Choi-Beutler- 2019 , Choi-Busino-2018 , Choudhury-Michlewski-2017 , Christianson-Kopito-2011 , Cloutier-Coulombe-2013 , Cloutier-Coulombe-2017 , Colicelli-2010 , Colland-  Gauthier-2004 , Conte-Perez-Oliva-2018 , Cooper-Green-2015 , Corominas-  Iakoucheva-2014 , Couzens-Gingras-2013 , Cox-Rizzino-2013 , Coyaud-Raught- 2015 , Crow-Cristea-2017  **D** |

Daakour-Twizere-2016 , Dabbaghizadeh-Tanguay-2018 , Dart-Wells-2015 , Das-

Broemer-2019 , Davis-Glaunsinger-2015 , de Hoog-Mann-2004 , Devarajan-Ketha- Kumar-2012 , Diner-Cristea-2015 , Dittmer-Misteli-2014 , Dobbin-Giordano-2005 ,

| Douanne-Bidère-2019 , Drissi-Boisvert-2015 , Du-Krogan-2017  **E** |
| --- |
| Elliott-Gyrd-Hansen-2016 , Emdal-Olsen-2015 , Enzo-Dupont-2015 , Ertych- Bastians-2016 , Ewing-Figeys-2007  **F** |
| Fang-Lin-2011 , Faust-Frankel-2018 , Fenner-Prehn-2010 , Floyd-Pagliarini-2016 , Foerster-Ritter-2013 , Fogeron-Lange-2013 , Fonseca-Damgaard-2015 , Foster-  Marshall-2013 , Fragoza-Yu-2019 , Freibaum-Taylor-2010  **G** |
| Gabriel-Baumgrass-2016 , Gallardo-Vara-Bernabeu-2019 , Galligan-Howley-2015 ,  Gao-Reinberg-2012 , Gao-Vaziri-2016 , Garzia-Sonenberg-2017 , Gautier-Hall-2009 , Giannone-Liu-2010 , Gilmore-Washburn-2016 , Giurato-Tarallo-2018 , Glatter-  Gstaiger-2009 , Gloeckner-Ueffing-2007 , Goehler-Wanker-2004 , Gordon-Krogan- 2020 , Goudreault-Gingras-2009 , Greco-Cristea-2011 , Grossmann-Stelzl-2015 ,  Guarani-Harper-2014 , Guard-Old-2019 , Guardia-Laguarta-Przedborski-2019 , Guderian-Grimmler-2011 , Gupta-Pelletier-2015  **H** |
| Han-Bassik-2017 A , Han-Bassik-2017 B , Hanson-Clayton-2014 , Hauri-Beisel-2016 , Hauri-Gstaiger-2013 , Havrylov-Redowicz-2009 , Havugimana-Emili-2012 , Hayes-  Urbé-2012 , Hegele-Stelzl-2012 A , Hegele-Stelzl-2012 B , Heidelberger-Beli-2018 , Hein-Mann-2015 , Hermjakob-Apweiler-2004 , Herr-Helleday-2015 , Hoffmeister-  Längst-2017 , Horlbeck-Gilbert-2018 A , Horlbeck-Gilbert-2018 B , Hosp-Selbach- 2015 , Hou-Chen-2018 , Hou-Huang-2017 , Hu-Woods-2019 , Hu-Yin-2019 , Hubel- Pichlmair-2019 , Huber-Hoelz-2017 , HUMANCYC , Humphries-Humphries-2009 , Hussain-Aldaz-2018 , Hutchins-Peters-2010 , Huttlin-Gygi-2015 , Huttlin-Harper- 2017 , Hüttenhain-Krogan-2019  **I** |

I2D-BIND-Fly2Human , I2D-BIND-Mouse2Human , I2D-BIND-Rat2Human , I2D- BIND-Worm2Human , I2D-BIND-Yeast2Human , I2D-BioGRID-Fly2Human , I2D- BioGRID-Mouse2Human , I2D-BioGRID-Rat2Human , I2D-BioGRID-

Worm2Human , I2D-BioGRID-Yeast2Human , I2D-Chen-Pawson-2009-PiwiScreen- Mouse2Human , I2D-Formstecher-Daviet-2005-Embryo-Fly2Human , I2D-

Formstecher-Daviet-2005-Head-Fly2Human , I2D-Giot-Rothbert-2003-High-

Fly2Human , I2D-Giot-Rothbert-2003-Low-Fly2Human , I2D-INNATEDB-

Mouse2Human , I2D-IntAct-Fly2Human , I2D-IntAct-Mouse2Human , I2D-IntAct- Rat2Human , I2D-IntAct-Worm2Human , I2D-IntAct-Yeast2Human , I2D-Krogan- Greenblatt-2006-Core-Yeast2Human , I2D-Krogan-Greenblatt-2006-NonCore-

Yeast2Human , I2D-Li-Vidal-2004-CE-DATA-Worm2Human , I2D-Li-Vidal-2004-

CORE-1-Worm2Human , I2D-Li-Vidal-2004-CORE-2-Worm2Human , I2D-Li-Vidal- 2004-interolog-Worm2Human , I2D-Li-Vidal-2004-literature-Worm2Human , I2D-Li-

| Vidal-2004-non-core-Worm2Human , I2D-Manual-Mouse2Human , I2D-Manual-  Rat2Human , I2D-MGI-Mouse2Human , I2D-MINT-Fly2Human , I2D-MINT-  Mouse2Human , I2D-MINT-Rat2Human , I2D-MINT-Worm2Human , I2D-MINT- Yeast2Human , I2D-MIPS-Yeast2Human , I2D-Ptacek-Snyder-2005-Yeast2Human , I2D-Stanyon-Finley-2004-CellCycle-Fly2Human , I2D-Tarassov-PCA-  Yeast2Human , I2D-Tewari-Vidal-2004-TGFb-Worm2Human , I2D-vonMering-  Bork-2002-High-Yeast2Human , I2D-vonMering-Bork-2002-Low-Yeast2Human ,  I2D-vonMering-Bork-2002-Medium-Yeast2Human , I2D-Wang-Orkin-2006-EScmplx- Mouse2Human , I2D-Wang-Orkin-2006-EScmplxIP-Mouse2Human , I2D-Wang-  Orkin-2006-EScmplxlow-Mouse2Human , I2D-Yu-Vidal-2008-GoldStd-  Yeast2Human , IMID , Ingham-Pawson-2005 , Innocenti-Brown-2011 ,  INTERPRO , Iradi-Borchelt-2018 , IREF-bhf-ucl , IREF-bind , IREF-bind-  translation , IREF-biogrid , IREF-corum , IREF-dip , IREF-hpidb , IREF-hprd , IREF-huri , IREF-innatedb , IREF-intact , IREF-intcomplex , IREF-matrixdb , IREF-mbinfo , IREF-mint , IREF-mppi , IREF-quickgo , IREF-reactome , IREF-  SMALL-SCALE-STUDIES , IREF-SMALL-SCALE-STUDIES , IREF-spike , IREF- uniprotpp , IREF-virushost , Ivanochko-Arrowsmith-2019  **J** |
| --- |
| Jain-Parker-2016 , Jang-Trono-2018 , Jeronimo-Coulombe-2007 , Jiang-de Kok- 2017 , Jin-Pawson-2004 , Jirawatnotai-Sicinski-2011 , Johnson-Kerner-Wichterle- 2015 , Johnson-Shoemaker-2003 , Jones-MacBeath-2006 , Joshi-Cristea-2013 ,  Jozwik-Carroll-2016 , Jäger-Krogan-2011  **K** |
| Kahle-Zoghbi-2011 , Kaltenbach-Hughes-2007 , Kang-Shin-2015 , Karras-Soengas- 2019 , Kato-Sternberg-2014 , Katsogiannou-Rocchi-2014 , Kawahara-Paes Leme-  2017 , Keller-Lee-2014 , Kennedy-Kolch-2020 A , Kennedy-Kolch-2020 B , Khanna- Parnaik-2018 , Kim-Major-2015 , Kneissl-Grummt-2003 , Koch-Hermeking-2007 ,  Kotlyar-Jurisica-2015 , Kristensen-Foster-2012 , Kumar-Maddika-2017 , Kumar- Vertegaal-2017 , Kupka-Walczak-2016 , Kärblane-Sarmiento-2015 , Kırlı-Görlich- 2015  **L** |

Lambert-Gingras-2015 , Lampert-Peter-2018 , Lau-Ronai-2012 , Lee-Choi-2016 ,

Lee-Choi-2017 , Lee-Jeong-2017 , Lee-Jou-2019 , Lee-Mayr-2019 , Lee-Songyang-

2011 , Lehner-Sanderson-2004 A , Lehner-Sanderson-2004 B , Leung-Jones-2014 ,

Leung-Miller-2017 , Li-Chen-2015 , Li-Dorf-2011 A , Li-Dorf-2011 B , Li-Dorf-2014 , Li-Fu-2017 , Li-Haura-2013 , Li-Hung-2019 , Li-Lu-2018 , Li-Wang-2016 , Li-Zhou- 2017 , Liebelt-Vertegaal-2020 , Lim-Zoghbi-2006 , Lin-Smith-2010 , Lipp-Guthrie-

2015 , Liu-Chen-2019 , Liu-Sun-2019 , Liu-Takahashi-2017 , Liu-Tan-2018 , Liu-

Varjosalo-2018 , Liu-Wang-2012 , Liu-Xu-2018 , Liu-Yang-2019 , Llères-Lamond-

2010 , Loch-Strickler-2012 , Low-Heck-2014 , Lu-Bohr-2017 , Lu-Zhang-2013 , Luck- Calderwood-2020 , Lum-Cristea-2018 , Luo-Elledge-2009

| Mak-Moffat-2010 , Malinová-Verheggen-2017 , Mallon-McKay-2013 , Malovannaya- Qin-2010 , Malty-Babu-2017 , Markson-Sanderson-2009 , Martin-Elledge-2017 ,  Maréchal-Zou-2014 , Matsumoto-Nakayama-2005 , Matsuoka-Elledge-2007 ,  McCracken-Blencowe-2005 , McFarland-Nussbaum-2008 , McNamara-D'Orso-2016 ,  Meek-Piwnica-Worms-2004 , Menon-Litovchick-2019 , Milev-Mouland-2012 ,  Miyamoto-Sato-Yanagawa-2010 , Mohammed-Carroll-2013 , Moon-Kim-2014 , Moutaoufik-Babu-2019 , Mugabo-Lim-2018 , Muller-Demeret-2012 , Murakawa- Landthaler-2015  **N** |
| --- |
| Nakamura-Groth-2019 , Nakayama-Ohara-2002 , Napolitano-Meroni-2011 ,  Narayan-Bennett-2012 , Nassa-Weisz-2019 , Nathan-Goldberg-2013 ,  NCI NATURE , Neganova-Lako-2011 , Newman-Keating-2003 , Noguchi- Kawahara-2018 , Nowak-Sommer-2019  **O** |
| Oliviero-Cagney-2015 , Oliviero-Cagney-2016 , Olma-Pintard-2009 , Oláh-Ovádi- 2011 , Ouyang-Gill-2009  **P** |
| Panigrahi-Pati-2012 , Pankow-Yates-2015 , Pao-Virdee-2018 , Papp-Lamia-2015 ,  Pech-Settleman-2019 , Perez-Hernandez-Yáñez-Mó-2013 , Perez-Perri-Espinosa-  2016 , Perou-Botstein-1999 , Perou-Botstein-2000 , Persaud-Rotin-2009 A , Persaud- Rotin-2009 B , Petschnigg-Stagljar-2014 , PFAM , Phillips-Corn-2013 , Pichlmair-  Superti-Furga-2011 , Pichlmair-Superti-Furga-2012 , Pilling-Cooper-2017 ,  Pladevall-Morera-Lopez-Contreras-2019 , Ptushkina-Ray-2017  **R** |
| Raisner-Gascoigne-2018 , Ramachandran-LaBaer-2004 , Raman-Harper-2015 ,  Ramaswamy-Golub-2001 , Ravasi-Hayashizaki-2010 , REACTOME , Reinke-  Keating-2010 , Reinke-Keating-2013 , Rengasamy-Walsh-2017 , Reyniers-Taymans- 2014 , Richter-Chrzanowska-Lightowlers-2010 , Rieger-Chu-2004 , Rivera-Paes  Leme-2018 , Rodriguez-von Kriegsheim-2016 , Roewenstrunk-de la Luna-2019 ,  Rolland-Vidal-2014 , Rosenbluh-Hahn-2016 , Rosenwald-Staudt-2001 , Ross-Perou- 2001 , Roth-Zlotnik-2006 , Rowbotham-Mermoud-2011 , Roy-Pardo-2014 , Roy-  Parent-2013 , Rual-Vidal-2005  **S** |

Saez-Vilchez-2018 , Sahni-Vidal-2015 , Saito-Kobarg-2017 , Sala-Ampe-2017 ,

Salvetti-Greco-2016 , Sang-Jackson-2011 , Sato-Conaway-2004 , Savidis-Brass-2016 , Schadt-Shoemaker-2004 , Schiza-Diamandis-2018 , Scholz-Taylor-2016 , Scifo-

Lalowski-2015 , Scott-Guy-2017 , Scott-Schulman-2016 , Shami Shah-Baskin-2019 , Shen-Chen-2019 , Shen-Mali-2017 , Sherman-Teitell-2010 , Simabuco-Zanchin-2019 , Singh-Moore-2012 , So-Colwill-2015 , Sokolina-Stagljar-2017 , Soler-López-Aloy-

2011 , Sowa-Harper-2009 , Srivas-Ideker-2016 , St-Denis-Gingras-2015 , St-Denis-

| Gingras-2016 , Stehling-Lill-2012 , Stehling-Lill-2013 , Stelzl-Wanker-2005 , Stuart- Kim-2003 , Sundell-Ivarsson-2018 , Suter-Wanker-2013 , Swayampakula-Dedhar-  2017  **T** |
| --- |
| Taipale-Lindquist-2012 , Taipale-Lindquist-2014 , Takahashi-Conaway-2011 , Tang- Wang-2019 , Tarallo-Weisz-2011 , Teixeira-Gomes-2010 , Teixeira-Laman-2016 A , Teixeira-Laman-2016 B , Thalappilly-Dusetti-2008 , Thompson-Luchansky-2014 ,  Tiemann-Kani-2019 , Tomkins-Manzoni-2018 , Tong-Moran-2014 , Toyoshima-  Grandori-2012 , Trepte-Wanker-2018 A , Trepte-Wanker-2018 B , Tsai-Cristea-2012  **U** |
| Ugidos-Vandenbroeck-2019  **V** |
| Van Acker-Dewilde-2019 , Van Alstyne-Pellizzoni-2018 , Van Quickelberghe- Gevaert-2018 , van Wijk-Timmers-2009 , Vandamme-Angrand-2011 , Varier- Vermeulen-2016 , Varjosalo-Gstaiger-2013 A , Varjosalo-Gstaiger-2013 B ,  Varjosalo-Superti-Furga-2013 , Vastrik-Stein-2007 , Venkatesan-Vidal-2009 , Viita- Vartiainen-2019 , Vinayagam-Wanker-2011 , Virok-Fülöp-2011 , Vizeacoumar-  Moffat-2013 , von Hundelshausen-Weber-2017  **W** |
| Wallach-Kramer-2013 , Wan-Emili-2015 , Wang-Balch-2006 , Wang-Cheung-2015 , Wang-He-2008 , Wang-Huang-2017 , Wang-Liu-2019 , Wang-Maris-2006 , Wang-  Xiong-2019 , Wang-Xu-2015 , Wang-Yang-2011 , Watanabe-Fujita-2018 , Weimann- Stelzl-2013 A , Weimann-Stelzl-2013 B , Weinmann-Meister-2009 , Weishäupl-  Schmidt-2019 , Weith-Meyer-2018 , Whisenant-Salomon-2015 , Wilkinson-Coba-  2019 , Willingham-Muchowski-2003 , Winczura-Jensen-2018 , Wong-O'Bryan-2012 , Woods-Monteiro-2012 A , Woods-Monteiro-2012 B , Woodsmith-Sanderson-2012 ,  Wu-Garvey-2007 , Wu-Li-2007 , Wu-Ma-2012 , Wu-Stein-2010 , Wu-Stein-2010  **X** |
| Xiao-Brown-2018 , Xiao-Lefkowitz-2007 , Xie-Cong-2013 , Xie-Green-2012 , Xie- Zhang-2017 , Xu-Ye-2012 , Xu-Zetter-2016  **Y** |
| Yachie-Roth-2016 , Yadav-Varjosalo-2017 , Yamauchi-Maeda-2018 , Yang-Brasier- 2015 , Yang-Chen-2010 , Yang-Maurer-2018 , Yang-Vidal-2016 , Yang-Wang-2018 , Yao-Stagljar-2017 A , Yao-Stagljar-2017 B , Yatim-Benkirane-2012 , Yeung-  Dougan-2019 , Yu-Chow-2013 , Yu-Engel-2018 , Yu-Vidal-2011 , Yue-Liu-2018  **Z** |

Zanon-Pichler-2013 , Zeller-Wei-2006 , Zhang-Shang-2006 , Zhang-Vermeulen-2017 , Zhang-Wang-2018 , Zhang-Wheeler-2014 , Zhang-Xu-2018 , Zhang-Zou-2011 , Zhao- Krug-2005 , Zhao-Yang-2011 , Zhong-Vidal-2016 , Zhou-Conrads-2004 , Zhou-

Hanemann-2016 , Zhu-Liu-2018

Genes

**Gene Description Rank**

| DERL3 | derlin 3 [Source:HGNC Symbol;Acc:HGNC:14236] | N/A |
| --- | --- | --- |
| NOD1 | nucleotide binding oligomerization domain containing 1 [Source:HGNC Symbol;Acc:HGNC:16390] | N/A |
| DNAJB9 | DnaJ heat shock protein family (Hsp40) member B9 [Source:HGNC Symbol;Acc:HGNC:6968] | N/A |
| SEL1L | SEL1L adaptor subunit of ERAD E3 ubiquitin ligase [Source:HGNC Symbol;Acc:HGNC:10717] | N/A |
| CFTR | CF transmembrane conductance regulator [Source:HGNC Symbol;Acc: HGNC:1884] | N/A |
| RIPK2 | receptor interacting serine/threonine kinase 2 [Source:HGNC Symbol;Acc: HGNC:10020] | 1 |
| DERL2 | derlin 2 [Source:HGNC Symbol;Acc:HGNC:17943] | 2 |
| DERL1 | derlin 1 [Source:HGNC Symbol;Acc:HGNC:28454] | 3 |
| SLC9A3R1 | SLC9A3 regulator 1 [Source:HGNC Symbol;Acc:HGNC:11075] | 4 |
| ERLEC1 | endoplasmic reticulum lectin 1 [Source:HGNC Symbol;Acc:HGNC:25222] | 5 |
| SLC4A7 | solute carrier family 4 member 7 [Source:HGNC Symbol;Acc:HGNC: 11033] | 6 |
| EVA1C | eva-1 homolog C [Source:HGNC Symbol;Acc:HGNC:13239] | 7 |
| SLC26A8 | solute carrier family 26 member 8 [Source:HGNC Symbol;Acc:HGNC: 14468] | 8 |
| NOTCH1 | notch receptor 1 [Source:HGNC Symbol;Acc:HGNC:7881] | 9 |
| XBP1 | X-box binding protein 1 [Source:HGNC Symbol;Acc:HGNC:12801] | 10 |
| UBE2J1 | ubiquitin conjugating enzyme E2 J1 [Source:HGNC Symbol;Acc:HGNC: 17598] | 11 |
| CARD9 | caspase recruitment domain family member 9 [Source:HGNC Symbol;Acc: HGNC:16391] | 12 |
| OS9 | OS9 endoplasmic reticulum lectin [Source:HGNC Symbol;Acc:HGNC: 16994] | 13 |
| GPS1 | G protein pathway suppressor 1 [Source:HGNC Symbol;Acc:HGNC:4549] | 14 |
| EDEM1 | ER degradation enhancing alpha-mannosidase like protein 1 [Source: HGNC Symbol;Acc:HGNC:18967] | 15 |
| HM13 | histocompatibility minor 13 [Source:HGNC Symbol;Acc:HGNC:16435] | 16 |
| DNAJC5 | DnaJ heat shock protein family (Hsp40) member C5 [Source:HGNC | 17 |

| Symbol;Acc:HGNC:16235] | | |
| --- | --- | --- |
| CARD6 | caspase recruitment domain family member 6 [Source:HGNC Symbol;Acc: HGNC:16394] | 18 |
| OLFM4 | olfactomedin 4 [Source:HGNC Symbol;Acc:HGNC:17190] | 19 |

AUP1 AUP1 lipid droplet regulating VLDL assembly factor [Source:HGNC 20

Symbol;Acc:HGNC:891]

Networks

**Physical Interactions** 77.64%

| Wang-Huang-2017  Molecular Details Underlying Dynamic Structures and Regulation of the Human 26S Proteasome. Wang et al (2017). *Mol Cell Proteomics*  Physical Interactions with 73 interactions from BioGRID | 4.41% |
| --- | --- |
| von Hundelshausen-Weber-2017  Chemokine interactome mapping enables tailored intervention in acute and chronic inflammation. von Hundelshausen et al  (2017). *Sci Transl Med*  Physical Interactions with 213 interactions from iRefIndex | 2.33% |
| McFarland-Nussbaum-2008  Proteomics analysis identifies phosphorylation-dependent alpha-synuclein protein interactions. McFarland et al (2008). *Mol Cell Proteomics*  Physical Interactions with 159 interactions from BioGRID | 2.21% |
| Guardia-Laguarta-Przedborski-2019  PINK1 Content in Mitochondria is Regulated by ER-Associated Degradation. Guardia-Laguarta et al (2019). *J Neurosci* Physical Interactions with 468 interactions from BioGRID | 1.66% |
| Maréchal-Zou-2014  PRP19 transforms into a sensor of RPA-ssDNA after DNA damage and drives ATR activation via a ubiquitin-mediated circuitry. Maréchal et al (2014). *Mol Cell*  Physical Interactions with 996 interactions from BioGRID | 1.46% |
| Singh-Moore-2012  The cellular EJC interactome reveals higher-order mRNP structure and an EJC-SR protein nexus. Singh et al (2012). *Cell* Physical Interactions with 235 interactions from iRefIndex | 1.40% |
| Whisenant-Salomon-2015  The Activation-Induced Assembly of an RNA/Protein Interactome Centered on the Splicing Factor U2AF2 Regulates Gene Expression in Human CD4 T Cells. Whisenant et al (2015). *PLoS One*  Physical Interactions with 237 interactions from BioGRID | 1.39% |
| Tsai-Cristea-2012  Functional proteomics establishes the interaction of SIRT7 with chromatin remodeling complexes and expands its role in regulation of RNA polymerase I transcription. Tsai et al (2012). *Mol Cell Proteomics*  Physical Interactions with 651 interactions from BioGRID | 1.30% |
| Kneissl-Grummt-2003  Interaction and assembly of murine pre-replicative complex proteins in yeast and mouse cells. Kneissl et al (2003). *J Mol Biol* Physical Interactions with 81 interactions from BioGRID | 1.26% |
| Zhang-Vermeulen-2017  An Interaction Landscape of Ubiquitin Signaling. Zhang et al (2017). *Mol Cell* Physical Interactions with 242 interactions from BioGRID | 1.20% |
| Narayan-Bennett-2012  Short-chain 3-hydroxyacyl-coenzyme A dehydrogenase associates with a protein super-complex integrating multiple metabolic | 1.16% |

Narayan-Bennett-2012

pathways. Narayan et al (2012). *PLoS One*

Physical Interactions with 109 interactions from BioGRID

| Li-Fu-2017  The OncoPPi network of cancer-focused protein-protein interactions to inform biological insights and therapeutic strategies. Li et al (2017). *Nat Commun*  Physical Interactions with 749 interactions from BioGRID | 1.13% |
| --- | --- |
| Li-Hung-2019  MET Inhibitors Promote Liver Tumor Evasion of the Immune Response by Stabilizing PDL1. Li et al (2019). *Gastroenterology* Physical Interactions with 179 interactions from BioGRID | 1.09% |
| Richter-Chrzanowska-Lightowlers-2010  A functional peptidyl-tRNA hydrolase, ICT1, has been recruited into the human mitochondrial ribosome. Richter et al (2010).  *EMBO J*  Physical Interactions with 204 interactions from BioGRID | 1.07% |
| Ertych-Bastians-2016  CHK2-BRCA1 tumor-suppressor axis restrains oncogenic Aurora-A kinase to ensure proper mitotic microtubule assembly. Ertych et al (2016). *Proc Natl Acad Sci U S A*  Physical Interactions with 486 interactions from BioGRID | 1.07% |
| Yu-Chow-2013  VCP phosphorylation-dependent interaction partners prevent apoptosis in Helicobacter pylori-infected gastric epithelial cells. Yu et al (2013). *PLoS One*  Physical Interactions with 280 interactions from BioGRID | 1.01% |
| Lee-Jou-2019  Parkinson's disease-associated LRRK2-G2019S mutant acts through regulation of SERCA activity to control ER stress in astrocytes. Lee et al (2019). *Acta Neuropathol Commun*  Physical Interactions with 218 interactions from BioGRID | 0.98% |
| Colicelli-2010  ABL tyrosine kinases: evolution of function, regulation, and specificity. Colicelli (2010). *Sci Signal* Physical Interactions with 147 interactions from iRefIndex | 0.95% |
| Matsumoto-Nakayama-2005  Large-scale analysis of the human ubiquitin-related proteome. Matsumoto et al (2005). *Proteomics* Physical Interactions with 311 interactions from BioGRID | 0.94% |
| Kotlyar-Jurisica-2015  In silico prediction of physical protein interactions and characterization of interactome orphans. Kotlyar et al (2015). *Nat Methods* Physical Interactions with 121 interactions from BioGRID | 0.94% |
| Petschnigg-Stagljar-2014  The mammalian-membrane two-hybrid assay (MaMTH) for probing membrane-protein interactions in human cells. Petschnigg et al (2014). *Nat Methods*  Physical Interactions with 116 interactions from iRefIndex | 0.92% |
| Guarani-Harper-2014  TIMMDC1/C3orf1 functions as a membrane-embedded mitochondrial complex I assembly factor through association with the | 0.89% |

Guarani-Harper-2014

MCIA complex. Guarani et al (2014). *Mol Cell Biol*

Physical Interactions with 323 interactions from BioGRID

| Lu-Zhang-2013  The HECT type ubiquitin ligase NEDL2 is degraded by anaphase-promoting complex/cyclosome (APC/C)-Cdh1, and its tight regulation maintains the metaphase to anaphase transition. Lu et al (2013). *J Biol Chem*  Physical Interactions with 186 interactions from iRefIndex | 0.81% |
| --- | --- |
| Berggård-James-2006  140 mouse brain proteins identified by Ca2+-calmodulin affinity chromatography and tandem mass spectrometry. Berggård et al  (2006). *J Proteome Res*  Physical Interactions with 151 interactions from BioGRID | 0.78% |
| Kupka-Walczak-2016  SPATA2-Mediated Binding of CYLD to HOIP Enables CYLD Recruitment to Signaling Complexes. Kupka et al (2016). *Cell Rep* Physical Interactions with 411 interactions from BioGRID | 0.75% |
| Freibaum-Taylor-2010  Global analysis of TDP-43 interacting proteins reveals strong association with RNA splicing and translation machinery. Freibaum et al (2010). *J Proteome Res*  Physical Interactions with 160 interactions from iRefIndex | 0.72% |
| Perez-Perri-Espinosa-2016  The TIP60 Complex Is a Conserved Coactivator of HIF1A. Perez-Perri et al (2016). *Cell Rep* Physical Interactions with 118 interactions from BioGRID | 0.65% |
| Jirawatnotai-Sicinski-2011  A function for cyclin D1 in DNA repair uncovered by protein interactome analyses in human cancers. Jirawatnotai et al (2011).  *Nature*  Physical Interactions with 122 interactions from BioGRID | 0.65% |
| Kırlı-Görlich-2015  A deep proteomics perspective on CRM1-mediated nuclear export and nucleocytoplasmic partitioning. Kırlı et al (2015). *Elife* Physical Interactions with 1,034 interactions from BioGRID | 0.65% |
| Leung-Jones-2014  Enhanced prediction of Src homology 2 (SH2) domain binding potentials using a fluorescence polarization-derived c-Met, c-Kit, ErbB, and androgen receptor interactome. Leung et al (2014). *Mol Cell Proteomics*  Physical Interactions with 199 interactions from iRefIndex | 0.62% |
| Yatim-Benkirane-2012  NOTCH1 nuclear interactome reveals key regulators of its transcriptional activity and oncogenic function. Yatim et al (2012).  *Mol Cell*  Physical Interactions with 127 interactions from BioGRID | 0.60% |
| Mak-Moffat-2010  A lentiviral functional proteomics approach identifies chromatin remodeling complexes important for the induction of pluripotency. Mak et al (2010). *Mol Cell Proteomics*  Physical Interactions with 110 interactions from BioGRID | 0.60% |
| Chen-Ge-2013 B | 0.60% |

Chen-Ge-2013 B

Bcl2-associated athanogene 3 interactome analysis reveals a new role in modulating proteasome activity. Chen et al (2013). *Mol Cell Proteomics*

Physical Interactions with 173 interactions from BioGRID

| Zanon-Pichler-2013  Profiling of Parkin-binding partners using tandem affinity purification. Zanon et al (2013). *PLoS One* Physical Interactions with 195 interactions from BioGRID | 0.59% |
| --- | --- |
| Raisner-Gascoigne-2018  Enhancer Activity Requires CBP/P300 Bromodomain-Dependent Histone H3K27 Acetylation. Raisner et al (2018). *Cell Rep* Physical Interactions with 174 interactions from BioGRID | 0.57% |
| Jang-Trono-2018  KAP1 facilitates reinstatement of heterochromatin after DNA replication. Jang et al (2018). *Nucleic Acids Res* Physical Interactions with 699 interactions from BioGRID | 0.57% |
| Ahn-Lee-2008  The tumour suppressor PTEN mediates a negative regulation of the E3 ubiquitin-protein ligase Nedd4. Ahn et al (2008). *Biochem J*  Physical Interactions with 105 interactions from BioGRID | 0.56% |
| IREF-reactome  Physical Interactions with 111,926 interactions from iRefIndex | 0.55% |
| Vastrik-Stein-2007  Reactome: a knowledge base of biologic pathways and processes. Vastrik et al (2007). *Genome Biol* Physical Interactions with 111,926 interactions from iRefIndex | 0.55% |
| Liu-Varjosalo-2018  An AP-MS- and BioID-compatible MAC-tag enables comprehensive mapping of protein interactions and subcellular localizations. Liu et al (2018). *Nat Commun*  Physical Interactions with 825 interactions from BioGRID | 0.50% |
| Wang-Liu-2019  POH1 contributes to hyperactivation of TGF- signaling and facilitates hepatocellular carcinoma metastasis through deubiquitinating TGF- receptors and caveolin-1. Wang et al (2019). *EBioMedicine*  Physical Interactions with 180 interactions from iRefIndex | 0.48% |
| Murakawa-Landthaler-2015  RC3H1 post-transcriptionally regulates A20 mRNA and modulates the activity of the IKK/NF- Bpathway. Murakawa et al  (2015). *Nat Commun*  Physical Interactions with 155 interactions from BioGRID | 0.47% |
| Elliott-Gyrd-Hansen-2016  SPATA2 Links CYLD to LUBAC, Activates CYLD, and Controls LUBAC Signaling. Elliott et al (2016). *Mol Cell* Physical Interactions with 543 interactions from BioGRID | 0.47% |
| Lee-Choi-2016  Interactomic analysis of REST/NRSF and implications of its functional links with the transcription suppressor TRIM28 during neuronal differentiation. Lee et al (2016). *Sci Rep*  Physical Interactions with 199 interactions from BioGRID | 0.46% |

Hüttenhain-Krogan-2019

0.46%

ARIH2 Is a Vif-Dependent Regulator of CUL5-Mediated APOBEC3G Degradation in HIV Infection. Hüttenhain et al (2019). *Cell Host Microbe*

Physical Interactions with 384 interactions from BioGRID

| IREF-bhf-ucl  Physical Interactions with 1,155 interactions from iRefIndex | 0.46% |
| --- | --- |
| Leung-Miller-2017  ZMYM3 regulates BRCA1 localization at damaged chromatin to promote DNA repair. Leung et al (2017). *Genes Dev* Physical Interactions with 247 interactions from BioGRID | 0.45% |
| Scifo-Lalowski-2015  Quantitative analysis of PPT1 interactome in human neuroblastoma cells. Scifo et al (2015). *Data Brief* Physical Interactions with 199 interactions from BioGRID | 0.45% |
| Koch-Hermeking-2007  Large-scale identification of c-MYC-associated proteins using a combined TAP/MudPIT approach. Koch et al (2007). *Cell Cycle* Physical Interactions with 207 interactions from BioGRID | 0.45% |
| Chen-Zhang-2013  Quantitative study of the interactome of PKC involved in the EGF-induced tumor cell chemotaxis. Chen et al (2013). *J Proteome Res*  Physical Interactions with 182 interactions from BioGRID | 0.43% |
| Neganova-Lako-2011  An important role for CDK2 in G1 to S checkpoint activation and DNA damage response in human embryonic stem cells. Neganova et al (2011). *Stem Cells*  Physical Interactions with 307 interactions from iRefIndex | 0.42% |
| Taipale-Lindquist-2012  Quantitative analysis of HSP90-client interactions reveals principles of substrate recognition. Taipale et al (2012). *Cell* Physical Interactions with 390 interactions from iRefIndex | 0.42% |
| Heidelberger-Beli-2018  Proteomic profiling of VCP substrates links VCP to K6-linked ubiquitylation and c-Myc function. Heidelberger et al (2018).  *EMBO Rep*  Physical Interactions with 1,347 interactions from BioGRID | 0.41% |
| Persaud-Rotin-2009 A  Comparison of substrate specificity of the ubiquitin ligases Nedd4 and Nedd4-2 using proteome arrays. Persaud et al (2009). *Mol Syst Biol*  Physical Interactions with 139 interactions from BioGRID | 0.39% |
| McNamara-D'Orso-2016  KAP1 Recruitment of the 7SK snRNP Complex to Promoters Enables Transcription Elongation by RNA Polymerase II. McNamara et al (2016). *Mol Cell*  Physical Interactions with 276 interactions from BioGRID | 0.38% |
| Xie-Cong-2013  Deubiquitinase FAM/USP9X interacts with the E3 ubiquitin ligase SMURF1 protein and protects it from ligase activity- dependent self-degradation. Xie et al (2013). *J Biol Chem* | 0.38% |

Xie-Cong-2013

Physical Interactions with 170 interactions from BioGRID

| Zhou-Conrads-2004  "An investigation into the human serum ""interactome""." Zhou et al (2004). *Electrophoresis* Physical Interactions with 112 interactions from iRefIndex | 0.38% |
| --- | --- |
| Alsulami-Cagney-2019  SETD1A Methyltransferase Is Physically and Functionally Linked to the DNA Damage Repair Protein RAD18. Alsulami et al  (2019). *Mol Cell Proteomics*  Physical Interactions with 288 interactions from BioGRID | 0.34% |
| Beyer-Boldt-2018  CRISPR/Cas9-mediated Genomic Editing of Cluap1/IFT38 Reveals a New Role in Actin Arrangement. Beyer et al (2018). *Mol Cell Proteomics*  Physical Interactions with 213 interactions from BioGRID | 0.34% |
| Fogeron-Lange-2013  LGALS3BP regulates centriole biogenesis and centrosome hypertrophy in cancer cells. Fogeron et al (2013). *Nat Commun* Physical Interactions with 1,492 interactions from BioGRID | 0.34% |
| Phillips-Corn-2013  Conformational dynamics control ubiquitin-deubiquitinase interactions and influence in vivo signaling. Phillips et al (2013). *Proc Natl Acad Sci U S A*  Physical Interactions with 142 interactions from BioGRID | 0.33% |
| IREF-hpidb  Physical Interactions with 166 interactions from iRefIndex | 0.33% |
| Diner-Cristea-2015  Interactions of the Antiviral Factor Interferon Gamma-Inducible Protein 16 (IFI16) Mediate Immune Signaling and Herpes Simplex Virus-1 Immunosuppression. Diner et al (2015). *Mol Cell Proteomics*  Physical Interactions with 334 interactions from BioGRID | 0.33% |
| Malovannaya-Qin-2010  Streamlined analysis schema for high-throughput identification of endogenous protein complexes. Malovannaya et al (2010). *Proc Natl Acad Sci U S A*  Physical Interactions with 299 interactions from BioGRID | 0.32% |
| Barr-Knapp-2009  Large-scale structural analysis of the classical human protein tyrosine phosphatome. Barr et al (2009). *Cell* Physical Interactions with 173 interactions from iRefIndex | 0.32% |
| Faust-Frankel-2018  The HIV-1 Tat protein recruits a ubiquitin ligase to reorganize the 7SK snRNP for transcriptional activation. Faust et al (2018).  *Elife*  Physical Interactions with 2,200 interactions from BioGRID | 0.32% |
| Pladevall-Morera-Lopez-Contreras-2019  Proteomic characterization of chromosomal common fragile site (CFS)-associated proteins uncovers ATRX as a regulator of CFS stability. Pladevall-Morera et al (2019). *Nucleic Acids Res*  Physical Interactions with 621 interactions from BioGRID | 0.32% |

Fenner-Prehn-2010

0.30%

Expanding the substantial interactome of NEMO using protein microarrays. Fenner et al (2010). *PLoS One* Physical Interactions with 103 interactions from iRefIndex

| Keller-Lee-2014  SAICAR induces protein kinase activity of PKM2 that is necessary for sustained proliferative signaling of cancer cells. Keller et al  (2014). *Mol Cell*  Physical Interactions with 147 interactions from iRefIndex | 0.29% |
| --- | --- |
| Persaud-Rotin-2009 B  Comparison of substrate specificity of the ubiquitin ligases Nedd4 and Nedd4-2 using proteome arrays. Persaud et al (2009). *Mol Syst Biol*  Physical Interactions with 155 interactions from BioGRID | 0.29% |
| Oliviero-Cagney-2016  Dynamic Protein Interactions of the Polycomb Repressive Complex 2 during Differentiation of Pluripotent Cells. Oliviero et al  (2016). *Mol Cell Proteomics*  Physical Interactions with 638 interactions from BioGRID | 0.29% |
| Nassa-Weisz-2019  The RNA-mediated estrogen receptor interactome of hormone-dependent human breast cancer cell nuclei. Nassa et al (2019). *Sci*  *Data*  Physical Interactions with 1,490 interactions from BioGRID | 0.29% |
| Weinmann-Meister-2009  Importin 8 is a gene silencing factor that targets argonaute proteins to distinct mRNAs. Weinmann et al (2009). *Cell* Physical Interactions with 96 interactions from BioGRID | 0.28% |
| Yang-Brasier-2015  Systematic Determination of Human Cyclin Dependent Kinase (CDK)-9 Interactome Identifies Novel Functions in RNA Splicing Mediated by the DEAD Box (DDX)-5/17 RNA Helicases. Yang et al (2015). *Mol Cell Proteomics*  Physical Interactions with 195 interactions from iRefIndex | 0.28% |
| Kristensen-Foster-2012  A high-throughput approach for measuring temporal changes in the interactome. Kristensen et al (2012). *Nat Methods* Physical Interactions with 7,044 interactions from BioGRID | 0.28% |
| Behzadnia-Lührmann-2007  Composition and three-dimensional EM structure of double affinity-purified, human prespliceosomal A complexes. Behzadnia et al  (2007). *EMBO J*  Physical Interactions with 107 interactions from iRefIndex | 0.27% |
| Llères-Lamond-2010  Direct interaction between hnRNP-M and CDC5L/PLRG1 proteins affects alternative splice site choice. Llères et al (2010).  *EMBO Rep*  Physical Interactions with 848 interactions from BioGRID | 0.26% |
| Weishäupl-Schmidt-2019  Physiological and pathophysiological characteristics of ataxin-3 isoforms. Weishäupl et al (2019). *J Biol Chem* Physical Interactions with 202 interactions from iRefIndex | 0.26% |
| Varjosalo-Gstaiger-2013 B | 0.25% |

Varjosalo-Gstaiger-2013 B

The protein interaction landscape of the human CMGC kinase group. Varjosalo et al (2013). *Cell Rep* Physical Interactions with 308 interactions from BioGRID

| Hein-Mann-2015  A human interactome in three quantitative dimensions organized by stoichiometries and abundances. Hein et al (2015). *Cell* Physical Interactions with 27,015 interactions from BioGRID | 0.24% |
| --- | --- |
| Hosp-Selbach-2015  Quantitative interaction proteomics of neurodegenerative disease proteins. Hosp et al (2015). *Cell Rep* Physical Interactions with 365 interactions from BioGRID | 0.24% |
| Hutchins-Peters-2010  Systematic analysis of human protein complexes identifies chromosome segregation proteins. Hutchins et al (2010). *Science* Physical Interactions with 1,783 interactions from BioGRID | 0.24% |
| Greco-Cristea-2011  Nuclear import of histone deacetylase 5 by requisite nuclear localization signal phosphorylation. Greco et al (2011). *Mol Cell Proteomics*  Physical Interactions with 256 interactions from BioGRID | 0.24% |
| Thompson-Luchansky-2014  Quantitative Lys- -Gly-Gly (diGly) proteomics coupled with inducible RNAi reveals ubiquitin-mediated proteolysis of DNA damage-inducible transcript 4 (DDIT4) by the E3 ligase HUWE1. Thompson et al (2014). *J Biol Chem*  Physical Interactions with 244 interactions from iRefIndex | 0.24% |
| Xiao-Lefkowitz-2007  Functional specialization of beta-arrestin interactions revealed by proteomic analysis. Xiao et al (2007). *Proc Natl Acad Sci U S A*  Physical Interactions with 404 interactions from BioGRID | 0.24% |
| Gloeckner-Ueffing-2007  A novel tandem affinity purification strategy for the efficient isolation and characterisation of native protein complexes. Gloeckner et al (2007). *Proteomics*  Physical Interactions with 100 interactions from BioGRID | 0.23% |
| Brehme-Superti-Furga-2009  Charting the molecular network of the drug target Bcr-Abl. Brehme et al (2009). *Proc Natl Acad Sci U S A* Physical Interactions with 626 interactions from BioGRID | 0.23% |
| Jones-MacBeath-2006  A quantitative protein interaction network for the ErbB receptors using protein microarrays. Jones et al (2006). *Nature* Physical Interactions with 158 interactions from iRefIndex | 0.23% |
| Scott-Schulman-2016  Two Distinct Types of E3 Ligases Work in Unison to Regulate Substrate Ubiquitylation. Scott et al (2016). *Cell* Physical Interactions with 111 interactions from BioGRID | 0.22% |
| Yang-Maurer-2018  rec-YnH enables simultaneous many-by-many detection of direct protein-protein and protein-RNA interactions. Yang et al (2018).  *Nat Commun*  Physical Interactions with 325 interactions from BioGRID | 0.22% |

| Xu-Zetter-2016  Prohibitin 1 regulates tumor cell apoptosis via the interaction with X-linked inhibitor of apoptosis protein. Xu et al (2016). *J Mol Cell Biol*  Physical Interactions with 719 interactions from BioGRID | 0.22% |
| --- | --- |
| IREF-quickgo  Physical Interactions with 9,985 interactions from iRefIndex | 0.22% |
| Van Quickelberghe-Gevaert-2018  A protein-protein interaction map of the TNF-induced NF- Bsignal transduction pathway. Van Quickelberghe et al (2018). *Sci Data*  Physical Interactions with 525 interactions from BioGRID | 0.22% |
| Mohammed-Carroll-2013  Endogenous purification reveals GREB1 as a key estrogen receptor regulatory factor. Mohammed et al (2013). *Cell Rep* Physical Interactions with 112 interactions from BioGRID | 0.22% |
| IREF-intcomplex  Physical Interactions with 212 interactions from iRefIndex | 0.22% |
| IREF-dip  Physical Interactions with 5,037 interactions from iRefIndex | 0.21% |
| Yang-Wang-2018  E3 Ligase Trim21 Ubiquitylates and Stabilizes Keratin 17 to Induce STAT3 Activation in Psoriasis. Yang et al (2018). *J Invest Dermatol*  Physical Interactions with 103 interactions from iRefIndex | 0.21% |
| Tiemann-Kani-2019  Loss of ER retention motif of AGR2 can impact mTORC signaling and promote cancer metastasis. Tiemann et al (2019).  *Oncogene*  Physical Interactions with 339 interactions from BioGRID | 0.21% |
| IREF-mbinfo  Physical Interactions with 113 interactions from iRefIndex | 0.21% |
| Wan-Emili-2015  Panorama of ancient metazoan macromolecular complexes. Wan et al (2015). *Nature* Physical Interactions with 16,627 interactions from BioGRID | 0.21% |
| Herr-Helleday-2015  A genome-wide IR-induced RAD51 foci RNAi screen identifies CDC73 involved in chromatin remodeling for DNA repair. Herr et al (2015). *Cell Discov*  Physical Interactions with 117 interactions from iRefIndex | 0.20% |
| Gupta-Pelletier-2015  A Dynamic Protein Interaction Landscape of the Human Centrosome-Cilium Interface. Gupta et al (2015). *Cell* Physical Interactions with 306 interactions from BioGRID | 0.20% |
| Havugimana-Emili-2012  A census of human soluble protein complexes. Havugimana et al (2012). *Cell* Physical Interactions with 13,651 interactions from BioGRID | 0.20% |

Kaltenbach-Hughes-2007

0.20%

Huntingtin interacting proteins are genetic modifiers of neurodegeneration. Kaltenbach et al (2007). *PLoS Genet* Physical Interactions with 101 interactions from iRefIndex

| Fonseca-Damgaard-2015  La-related Protein 1 (LARP1) Represses Terminal Oligopyrimidine (TOP) mRNA Translation Downstream of mTOR Complex 1 (mTORC1). Fonseca et al (2015). *J Biol Chem*  Physical Interactions with 105 interactions from BioGRID | 0.19% |
| --- | --- |
| Devarajan-Ketha-Kumar-2012  The sclerostin-bone protein interactome. Devarajan-Ketha et al (2012). *Biochem Biophys Res Commun* Physical Interactions with 99 interactions from BioGRID | 0.19% |
| Tang-Wang-2019  The p300/YY1/miR-500a-5p/HDAC2 signalling axis regulates cell proliferation in human colorectal cancer. Tang et al (2019).  *Nat Commun*  Physical Interactions with 294 interactions from BioGRID | 0.19% |
| Barrios-Rodiles-Wrana-2005  High-throughput mapping of a dynamic signaling network in mammalian cells. Barrios-Rodiles et al (2005). *Science* Physical Interactions with 596 interactions from iRefIndex | 0.19% |
| Salvetti-Greco-2016  Nuclear Functions of Nucleolin through Global Proteomics and Interactomic Approaches. Salvetti et al (2016). *J Proteome Res* Physical Interactions with 144 interactions from BioGRID | 0.19% |
| Malinová-Verheggen-2017  Assembly of the U5 snRNP component PRPF8 is controlled by the HSP90/R2TP chaperones. Malinová et al (2017). *J Cell Biol* Physical Interactions with 2,778 interactions from BioGRID | 0.19% |
| Jeronimo-Coulombe-2007  Systematic analysis of the protein interaction network for the human transcription machinery reveals the identity of the 7SK capping enzyme. Jeronimo et al (2007). *Mol Cell*  Physical Interactions with 699 interactions from BioGRID | 0.19% |
| Choudhury-Michlewski-2017  RNA-binding activity of TRIM25 is mediated by its PRY/SPRY domain and is required for ubiquitination. Choudhury et al  (2017). *BMC Biol*  Physical Interactions with 250 interactions from BioGRID | 0.19% |
| Lu-Bohr-2017  Cell cycle-dependent phosphorylation regulates RECQL4 pathway choice and ubiquitination in DNA double-strand break repair. Lu et al (2017). *Nat Commun*  Physical Interactions with 1,312 interactions from BioGRID | 0.19% |
| Hubel-Pichlmair-2019  A protein-interaction network of interferon-stimulated genes extends the innate immune system landscape. Hubel et al (2019). *Nat Immunol*  Physical Interactions with 2,707 interactions from BioGRID | 0.19% |
| Moon-Kim-2014  Interactome analysis of AMP-activated protein kinase (AMPK)- 1and - 1in INS-1 pancreatic beta-cells by affinity purification- | 0.18% |

Moon-Kim-2014

mass spectrometry. Moon et al (2014). *Sci Rep*

Physical Interactions with 171 interactions from BioGRID

| Woods-Monteiro-2012 A  Charting the landscape of tandem BRCT domain-mediated protein interactions. Woods et al (2012). *Sci Signal* Physical Interactions with 602 interactions from BioGRID | 0.18% |
| --- | --- |
| Rowbotham-Mermoud-2011  Maintenance of silent chromatin through replication requires SWI/SNF-like chromatin remodeler SMARCAD1. Rowbotham et al  (2011). *Mol Cell*  Physical Interactions with 114 interactions from BioGRID | 0.18% |
| Loch-Strickler-2012  A microarray of ubiquitylated proteins for profiling deubiquitylase activity reveals the critical roles of both chain and substrate. Loch et al (2012). *Biochim Biophys Acta*  Physical Interactions with 145 interactions from BioGRID | 0.18% |
| Rosenbluh-Hahn-2016  Genetic and Proteomic Interrogation of Lower Confidence Candidate Genes Reveals Signaling Networks in -Catenin-Active Cancers. Rosenbluh et al (2016). *Cell Syst*  Physical Interactions with 3,482 interactions from BioGRID | 0.18% |
| Liebelt-Vertegaal-2020  Transcription-coupled nucleotide excision repair is coordinated by ubiquitin and SUMO in response to ultraviolet irradiation. Liebelt et al (2020). *Nucleic Acids Res*  Physical Interactions with 100 interactions from BioGRID | 0.18% |
| Noguchi-Kawahara-2018  ZFP36L2 is a cell cycle-regulated CCCH protein necessary for DNA lesion-induced S-phase arrest. Noguchi et al (2018). *Biol Open*  Physical Interactions with 143 interactions from iRefIndex | 0.18% |
| Gilmore-Washburn-2016  WDR76 Co-Localizes with Heterochromatin Related Proteins and Rapidly Responds to DNA Damage. Gilmore et al (2016). *PLoS One*  Physical Interactions with 944 interactions from BioGRID | 0.18% |
| Agrawal-Sedivy-2010  Proteomic profiling of Myc-associated proteins. Agrawal et al (2010). *Cell Cycle* Physical Interactions with 105 interactions from BioGRID | 0.17% |
| Cai-Conaway-2007  YY1 functions with INO80 to activate transcription. Cai et al (2007). *Nat Struct Mol Biol* Physical Interactions with 106 interactions from BioGRID | 0.17% |
| Lee-Mayr-2019  Gain of Additional BIRC3 Protein Functions through 3'-UTR-Mediated Protein Complex Formation. Lee et al (2019). *Mol Cell* Physical Interactions with 1,759 interactions from BioGRID | 0.17% |
| Lee-Jeong-2017  FGF11 induced by hypoxia interacts with HIF-1 and enhances its stability. Lee et al (2017). *FEBS Lett* | 0.17% |

Lee-Jeong-2017

Physical Interactions with 100 interactions from iRefIndex

| Oláh-Ovádi-2011  Interactions of pathological hallmark proteins: tubulin polymerization promoting protein/p25, beta-amyloid, and alpha-synuclein. Oláh et al (2011). *J Biol Chem*  Physical Interactions with 1,854 interactions from BioGRID | 0.17% |
| --- | --- |
| Roewenstrunk-de la Luna-2019  A comprehensive proteomics-based interaction screen that links DYRK1A to RNF169 and to the DNA damage response. Roewenstrunk et al (2019). *Sci Rep*  Physical Interactions with 116 interactions from BioGRID | 0.17% |
| Mugabo-Lim-2018  Elucidation of the 14-3-3 interactome reveals critical roles of RNA-splicing factors during adipogenesis. Mugabo et al (2018). *J Biol Chem*  Physical Interactions with 111 interactions from BioGRID | 0.17% |
| Shami Shah-Baskin-2019  PLEKHA4/kramer Attenuates Dishevelled Ubiquitination to Modulate Wnt and Planar Cell Polarity Signaling. Shami Shah et al  (2019). *Cell Rep*  Physical Interactions with 2,927 interactions from BioGRID | 0.16% |
| Yao-Stagljar-2017 A  A Global Analysis of the Receptor Tyrosine Kinase-Protein Phosphatase Interactome. Yao et al (2017). *Mol Cell* Physical Interactions with 312 interactions from BioGRID | 0.16% |
| Emdal-Olsen-2015  Temporal proteomics of NGF-TrkA signaling identifies an inhibitory role for the E3 ligase Cbl-b in neuroblastoma cell differentiation. Emdal et al (2015). *Sci Signal*  Physical Interactions with 1,917 interactions from BioGRID | 0.16% |
| Zhang-Wheeler-2014  Progesterone receptor membrane component 1 is a functional part of the glucagon-like peptide-1 (GLP-1) receptor complex in pancreatic cells. Zhang et al (2014). *Mol Cell Proteomics*  Physical Interactions with 93 interactions from BioGRID | 0.16% |
| Tong-Moran-2014  Proteomic analysis of the epidermal growth factor receptor (EGFR) interactome and post-translational modifications associated with receptor endocytosis in response to EGF and stress. Tong et al (2014). *Mol Cell Proteomics*  Physical Interactions with 321 interactions from BioGRID | 0.16% |
| Yue-Liu-2018  VIRMA mediates preferential m<sup>6</sup>A mRNA methylation in 3'UTR and near stop codon and associates with alternative polyadenylation. Yue et al (2018). *Cell Discov*  Physical Interactions with 1,502 interactions from BioGRID | 0.16% |
| Papp-Lamia-2015  DNA damage shifts circadian clock time via Hausp-dependent Cry1 stabilization. Papp et al (2015). *Elife* Physical Interactions with 158 interactions from iRefIndex | 0.15% |

Cheng-DeCaprio-2017

Merkel cell polyomavirus recruits MYCL to the EP400 complex to promote oncogenesis. Cheng et al (2017). *PLoS Pathog*

0.15%

Cheng-DeCaprio-2017

Physical Interactions with 95 interactions from BioGRID

| Ramachandran-LaBaer-2004  Self-assembling protein microarrays. Ramachandran et al (2004). *Science* Physical Interactions with 123 interactions from iRefIndex | 0.15% |
| --- | --- |
| Low-Heck-2014  A systems-wide screen identifies substrates of the SCF TrCP ubiquitin ligase. Low et al (2014). *Sci Signal* Physical Interactions with 221 interactions from BioGRID | 0.15% |
| Liu-Takahashi-2017  TTF-1/NKX2-1 binds to DDB1 and confers replication stress resistance to lung adenocarcinomas. Liu et al (2017). *Oncogene* Physical Interactions with 105 interactions from iRefIndex | 0.15% |
| Roy-Pardo-2014  hnRNPA1 couples nuclear export and translation of specific mRNAs downstream of FGF-2/S6K2 signalling. Roy et al (2014).  *Nucleic Acids Res*  Physical Interactions with 386 interactions from BioGRID | 0.15% |
| Li-Dorf-2011 A  Mapping a dynamic innate immunity protein interaction network regulating type I interferon production. Li et al (2011).  *Immunity*  Physical Interactions with 400 interactions from BioGRID | 0.15% |
| Giannone-Liu-2010  The protein network surrounding the human telomere repeat binding factors TRF1, TRF2, and POT1. Giannone et al (2010).  *PLoS One*  Physical Interactions with 288 interactions from BioGRID | 0.14% |
| Choi-Busino-2018  PTPN14 regulates Roquin2 stability by tyrosine dephosphorylation. Choi et al (2018). *Cell Cycle* Physical Interactions with 1,087 interactions from BioGRID | 0.14% |
| Meek-Piwnica-Worms-2004  Comprehensive proteomic analysis of interphase and mitotic 14-3-3-binding proteins. Meek et al (2004). *J Biol Chem* Physical Interactions with 328 interactions from iRefIndex | 0.14% |
| Wang-Balch-2006  Hsp90 cochaperone Aha1 downregulation rescues misfolding of CFTR in cystic fibrosis. Wang et al (2006). *Cell* Physical Interactions with 200 interactions from BioGRID | 0.14% |
| Lum-Cristea-2018  Interactome and Proteome Dynamics Uncover Immune Modulatory Associations of the Pathogen Sensing Factor cGAS. Lum et al  (2018). *Cell Syst*  Physical Interactions with 165 interactions from iRefIndex | 0.14% |
| IREF-uniprotpp  Physical Interactions with 2,470 interactions from iRefIndex | 0.14% |
| Hu-Yin-2019  Poly(ADP-ribosyl)ation of BRD7 by PARP1 confers resistance to DNA-damaging chemotherapeutic agents. Hu et al (2019). | 0.14% |

Hu-Yin-2019

*EMBO Rep*

Physical Interactions with 479 interactions from BioGRID

| BIOGRID-SMALL-SCALE-STUDIES  Physical Interactions with 79,201 interactions from BioGRID | 0.14% |
| --- | --- |
| Wallach-Kramer-2013  Dynamic circadian protein-protein interaction networks predict temporal organization of cellular functions. Wallach et al (2013).  *PLoS Genet*  Physical Interactions with 143 interactions from BioGRID | 0.14% |
| Wu-Li-2007  Systematic identification of SH3 domain-mediated human protein-protein interactions by peptide array target screening. Wu et al  (2007). *Proteomics*  Physical Interactions with 1,105 interactions from iRefIndex | 0.14% |
| Floyd-Pagliarini-2016  Mitochondrial Protein Interaction Mapping Identifies Regulators of Respiratory Chain Function. Floyd et al (2016). *Mol Cell* Physical Interactions with 1,508 interactions from BioGRID | 0.14% |
| Moutaoufik-Babu-2019  Rewiring of the Human Mitochondrial Interactome during Neuronal Reprogramming Reveals Regulators of the Respirasome and Neurogenesis. Moutaoufik et al (2019). *iScience*  Physical Interactions with 6,357 interactions from BioGRID | 0.13% |
| Sowa-Harper-2009  Defining the human deubiquitinating enzyme interaction landscape. Sowa et al (2009). *Cell* Physical Interactions with 1,509 interactions from BioGRID | 0.13% |
| Woodsmith-Sanderson-2012  Systematic analysis of dimeric E3-RING interactions reveals increased combinatorial complexity in human ubiquitination networks. Woodsmith et al (2012). *Mol Cell Proteomics*  Physical Interactions with 206 interactions from iRefIndex | 0.13% |
| IREF-innatedb  Physical Interactions with 2,355 interactions from iRefIndex | 0.13% |
| IREF-mppi  Physical Interactions with 304 interactions from iRefIndex | 0.13% |
| Bennett-Harper-2010  Dynamics of cullin-RING ubiquitin ligase network revealed by systematic quantitative proteomics. Bennett et al (2010). *Cell* Physical Interactions with 4,362 interactions from BioGRID | 0.13% |
| Humphries-Humphries-2009  Proteomic analysis of integrin-associated complexes identifies RCC2 as a dual regulator of Rac1 and Arf6. Humphries et al (2009).  *Sci Signal*  Physical Interactions with 1,060 interactions from BioGRID | 0.13% |
| McCracken-Blencowe-2005  Proteomic analysis of SRm160-containing complexes reveals a conserved association with cohesin. McCracken et al (2005). *J Biol* | 0.12% |

McCracken-Blencowe-2005

*Chem*

Physical Interactions with 198 interactions from BioGRID

| Boldt-Roepman-2016  An organelle-specific protein landscape identifies novel diseases and molecular mechanisms. Boldt et al (2016). *Nat Commun* Physical Interactions with 4,898 interactions from BioGRID | 0.12% |
| --- | --- |
| Crow-Cristea-2017  Human Antiviral Protein IFIX Suppresses Viral Gene Expression during Herpes Simplex Virus 1 (HSV-1) Infection and Is Counteracted by Virus-induced Proteasomal Degradation. Crow et al (2017). *Mol Cell Proteomics*  Physical Interactions with 180 interactions from iRefIndex | 0.12% |
| Alexander-Wang-2018  Ubiquilin 2 modulates ALS/FTD-linked FUS-RNA complex dynamics and stress granule formation. Alexander et al (2018). *Proc Natl Acad Sci U S A*  Physical Interactions with 189 interactions from iRefIndex | 0.12% |
| Arroyo-Aloy-2014  Charting the molecular links between driver and susceptibility genes in colorectal cancer. Arroyo et al (2014). *Biochem Biophys Res Commun*  Physical Interactions with 621 interactions from iRefIndex | 0.12% |
| Bett-Hay-2013  The P-body component USP52/PAN2 is a novel regulator of HIF1A mRNA stability. Bett et al (2013). *Biochem J* Physical Interactions with 238 interactions from iRefIndex | 0.12% |
| Cox-Rizzino-2013  The SOX2-interactome in brain cancer cells identifies the requirement of MSI2 and USP9X for the growth of brain tumor cells. Cox et al (2013). *PLoS One*  Physical Interactions with 190 interactions from iRefIndex | 0.12% |
| Pao-Virdee-2018  Activity-based E3 ligase profiling uncovers an E3 ligase with esterification activity. Pao et al (2018). *Nature* Physical Interactions with 134 interactions from BioGRID | 0.11% |
| Bandyopadhyay-Ideker-2010  A human MAP kinase interactome. Bandyopadhyay et al (2010). *Nat Methods* Physical Interactions with 653 interactions from iRefIndex | 0.11% |
| Zhao-Krug-2005  Human ISG15 conjugation targets both IFN-induced and constitutively expressed proteins functioning in diverse cellular pathways. Zhao et al (2005). *Proc Natl Acad Sci U S A*  Physical Interactions with 140 interactions from iRefIndex | 0.11% |
| Enzo-Dupont-2015  Aerobic glycolysis tunes YAP/TAZ transcriptional activity. Enzo et al (2015). *EMBO J* Physical Interactions with 156 interactions from BioGRID | 0.10% |
| Huttlin-Harper-2017  Architecture of the human interactome defines protein communities and disease networks. Huttlin et al (2017). *Nature* Physical Interactions with 55,868 interactions from BioGRID | 0.10% |

Napolitano-Meroni-2011 0.10%

Functional interactions between ubiquitin E2 enzymes and TRIM proteins. Napolitano et al (2011). *Biochem J* Physical Interactions with 81 interactions from BioGRID

| Varjosalo-Superti-Furga-2013  Interlaboratory reproducibility of large-scale human protein-complex analysis by standardized AP-MS. Varjosalo et al (2013). *Nat Methods*  Physical Interactions with 484 interactions from BioGRID | 0.10% |
| --- | --- |
| Chi-Reed-2018  Interactome analyses revealed that the U1 snRNP machinery overlaps extensively with the RNAP II machinery and contains multiple ALS/SMA-causative proteins. Chi et al (2018). *Sci Rep*  Physical Interactions with 456 interactions from BioGRID | 0.10% |
| Li-Lu-2018  Heterozygous deletion of chromosome 17p renders prostate cancer vulnerable to inhibition of RNA polymerase II. Li et al (2018).  *Nat Commun*  Physical Interactions with 176 interactions from iRefIndex | 0.10% |
| Viita-Vartiainen-2019  Nuclear actin interactome analysis links actin to KAT14 histone acetyl transferase and mRNA splicing. Viita et al (2019). *J Cell Sci*  Physical Interactions with 213 interactions from BioGRID | 0.10% |
| Banks-Washburn-2016  TNIP2 is a Hub Protein in the NF- BNetwork with Both Protein and RNA Mediated Interactions. Banks et al (2016). *Mol Cell Proteomics*  Physical Interactions with 616 interactions from BioGRID | 0.09% |
| Tarallo-Weisz-2011  Identification of proteins associated with ligand-activated estrogen receptor in human breast cancer cell nuclei by tandem  affinity purification and nano LC-MS/MS. Tarallo et al (2011). *Proteomics* Physical Interactions with 244 interactions from BioGRID | 0.09% |
| Yamauchi-Maeda-2018  Genome-wide CRISPR-Cas9 Screen Identifies Leukemia-Specific Dependence on a Pre-mRNA Metabolic Pathway Regulated by DCPS. Yamauchi et al (2018). *Cancer Cell*  Physical Interactions with 261 interactions from iRefIndex | 0.09% |
| Douanne-Bidère-2019  CYLD Regulates Centriolar Satellites Proteostasis by Counteracting the E3 Ligase MIB1. Douanne et al (2019). *Cell Rep* Physical Interactions with 111 interactions from iRefIndex | 0.09% |
| Panigrahi-Pati-2012  A cohesin-RAD21 interactome. Panigrahi et al (2012). *Biochem J* Physical Interactions with 137 interactions from iRefIndex | 0.09% |
| Joshi-Cristea-2013  The functional interactome landscape of the human histone deacetylase family. Joshi et al (2013). *Mol Syst Biol* Physical Interactions with 375 interactions from BioGRID | 0.09% |
| Katsogiannou-Rocchi-2014 | 0.09% |

Katsogiannou-Rocchi-2014

The functional landscape of Hsp27 reveals new cellular processes such as DNA repair and alternative splicing and proposes novel anticancer targets. Katsogiannou et al (2014). *Mol Cell Proteomics*

Physical Interactions with 221 interactions from iRefIndex

| Trepte-Wanker-2018 A  LuTHy: a double-readout bioluminescence-based two-hybrid technology for quantitative mapping of protein-protein interactions in mammalian cells. Trepte et al (2018). *Mol Syst Biol*  Physical Interactions with 70 interactions from BioGRID | 0.09% |
| --- | --- |
| Lau-Ronai-2012  PKC promotes oncogenic functions of ATF2 in the nucleus while blocking its apoptotic function at mitochondria. Lau et al  (2012). *Cell*  Physical Interactions with 102 interactions from iRefIndex | 0.09% |
| Campos-Reinberg-2015  Analysis of the Histone H3.1 Interactome: A Suitable Chaperone for the Right Event. Campos et al (2015). *Mol Cell* Physical Interactions with 105 interactions from iRefIndex | 0.09% |
| Malty-Babu-2017  A Map of Human Mitochondrial Protein Interactions Linked to Neurodegeneration Reveals New Mechanisms of Redox Homeostasis and NF- BSignaling. Malty et al (2017). *Cell Syst*  Physical Interactions with 1,969 interactions from BioGRID | 0.09% |
| Roy-Parent-2013  Novel, gel-free proteomics approach identifies RNF5 and JAMP as modulators of GPCR stability. Roy et al (2013). *Mol Endocrinol*  Physical Interactions with 114 interactions from iRefIndex | 0.09% |
| Christianson-Kopito-2011  Defining human ERAD networks through an integrative mapping strategy. Christianson et al (2011). *Nat Cell Biol* Physical Interactions with 294 interactions from BioGRID | 0.09% |
| Li-Dorf-2014  TRIM65 regulates microRNA activity by ubiquitination of TNRC6. Li et al (2014). *Proc Natl Acad Sci U S A* Physical Interactions with 495 interactions from BioGRID | 0.09% |
| Behrends-Harper-2010  Network organization of the human autophagy system. Behrends et al (2010). *Nature* Physical Interactions with 704 interactions from BioGRID | 0.09% |
| Drissi-Boisvert-2015  Quantitative Proteomics Reveals Dynamic Interactions of the Minichromosome Maintenance Complex (MCM) in the Cellular Response to Etoposide Induced DNA Damage. Drissi et al (2015). *Mol Cell Proteomics*  Physical Interactions with 947 interactions from BioGRID | 0.09% |
| Foerster-Ritter-2013  Characterization of the EGFR interactome reveals associated protein complex networks and intracellular receptor dynamics. Foerster et al (2013). *Proteomics*  Physical Interactions with 179 interactions from BioGRID | 0.08% |
| Li-Chen-2015 | 0.08% |

Li-Chen-2015

Proteomic analyses reveal distinct chromatin-associated and soluble transcription factor complexes. Li et al (2015). *Mol Syst Biol* Physical Interactions with 1,811 interactions from BioGRID

| Lipp-Guthrie-2015  SR protein kinases promote splicing of nonconsensus introns. Lipp et al (2015). *Nat Struct Mol Biol* Physical Interactions with 388 interactions from BioGRID | 0.08% |
| --- | --- |
| IREF-mint  Physical Interactions with 14,408 interactions from iRefIndex | 0.08% |
| Kennedy-Kolch-2020 A  Extensive rewiring of the EGFR network in colorectal cancer cells expressing transforming levels of KRAS<sup>G13D</sup>. Kennedy et al (2020). *Nat Commun*  Physical Interactions with 4,232 interactions from BioGRID | 0.08% |
| IREF-corum  Physical Interactions with 819 interactions from iRefIndex | 0.08% |
| Kärblane-Sarmiento-2015  ABCE1 is a highly conserved RNA silencing suppressor. Kärblane et al (2015). *PLoS One* Physical Interactions with 140 interactions from iRefIndex | 0.07% |
| Wong-O'Bryan-2012  Intersectin (ITSN) family of scaffolds function as molecular hubs in protein interaction networks. Wong et al (2012). *PLoS One* Physical Interactions with 114 interactions from BioGRID | 0.07% |
| Li-Zhou-2017  Identification of translationally controlled tumor protein in promotion of DNA homologous recombination repair in cancer cells by affinity proteomics. Li et al (2017). *Oncogene*  Physical Interactions with 104 interactions from iRefIndex | 0.07% |
| Wang-Xu-2015  Interaction of amyotrophic lateral sclerosis/frontotemporal lobar degeneration-associated fused-in-sarcoma with proteins involved in metabolic and protein degradation pathways. Wang et al (2015). *Neurobiol Aging*  Physical Interactions with 197 interactions from BioGRID | 0.07% |
| Saito-Kobarg-2017  Human Regulatory Protein Ki-1/57 Is a Target of SUMOylation and Affects PML Nuclear Body Formation. Saito et al (2017). *J Proteome Res*  Physical Interactions with 165 interactions from iRefIndex | 0.07% |
| Dabbaghizadeh-Tanguay-2018  Identification of proteins interacting with the mitochondrial small heat shock protein Hsp22 of Drosophila melanogaster: Implication in mitochondrial homeostasis. Dabbaghizadeh et al (2018). *PLoS One*  Physical Interactions with 144 interactions from BioGRID | 0.07% |
| Zhang-Xu-2018  Revealing A-Raf functions through its interactome. Zhang et al (2018). *Biochim Biophys Acta Proteins Proteom* Physical Interactions with 186 interactions from BioGRID | 0.07% |
| Hauri-Beisel-2016 | 0.07% |

Hauri-Beisel-2016

A High-Density Map for Navigating the Human Polycomb Complexome. Hauri et al (2016). *Cell Rep* Physical Interactions with 1,216 interactions from BioGRID

| Ewing-Figeys-2007  Large-scale mapping of human protein-protein interactions by mass spectrometry. Ewing et al (2007). *Mol Syst Biol* Physical Interactions with 5,759 interactions from iRefIndex | 0.07% |
| --- | --- |
| Menon-Litovchick-2019  DYRK1A regulates the recruitment of 53BP1 to the sites of DNA damage in part through interaction with RNF169. Menon et al  (2019). *Cell Cycle*  Physical Interactions with 119 interactions from BioGRID | 0.07% |
| IREF-bind-translation  Physical Interactions with 6,056 interactions from iRefIndex | 0.07% |
| Hayes-Urbé-2012  Direct and indirect control of mitogen-activated protein kinase pathway-associated components, BRAP/IMP E3 ubiquitin ligase and CRAF/RAF1 kinase, by the deubiquitylating enzyme USP15. Hayes et al (2012). *J Biol Chem*  Physical Interactions with 110 interactions from BioGRID | 0.06% |
| Reyniers-Taymans-2014  Differential protein-protein interactions of LRRK1 and LRRK2 indicate roles in distinct cellular signaling pathways. Reyniers et al (2014). *J Neurochem*  Physical Interactions with 109 interactions from iRefIndex | 0.06% |
| Botham-Schimmer-2019  Global Interactome Mapping of Mitochondrial Intermembrane Space Proteases Identifies a Novel Function for HTRA2. Botham et al (2019). *Proteomics*  Physical Interactions with 317 interactions from BioGRID | 0.06% |
| Kim-Major-2015  Substrate trapping proteomics reveals targets of the TrCP2/FBXW11 ubiquitin ligase. Kim et al (2015). *Mol Cell Biol* Physical Interactions with 137 interactions from BioGRID | 0.06% |
| Grossmann-Stelzl-2015  Phospho-tyrosine dependent protein-protein interaction network. Grossmann et al (2015). *Mol Syst Biol* Physical Interactions with 620 interactions from BioGRID | 0.06% |
| IREF-bind  Physical Interactions with 3,524 interactions from iRefIndex | 0.06% |
| Bouwmeester-Superti-Furga-2004  A physical and functional map of the human TNF-alpha/NF-kappa B signal transduction pathway. Bouwmeester et al (2004).  *Nat Cell Biol*  Physical Interactions with 1,694 interactions from iRefIndex | 0.06% |
| St-Denis-Gingras-2015  Myotubularin-related proteins 3 and 4 interact with polo-like kinase 1 and centrosomal protein of 55 kDa to ensure proper abscission. St-Denis et al (2015). *Mol Cell Proteomics*  Physical Interactions with 168 interactions from BioGRID | 0.05% |

0.05%

Blomen-Brummelkamp-2015

Gene essentiality and synthetic lethality in haploid human cells. Blomen et al (2015). *Science* Physical Interactions with 138 interactions from BioGRID

| Li-Haura-2013  Perturbation of the mutated EGFR interactome identifies vulnerabilities and resistance mechanisms. Li et al (2013). *Mol Syst Biol*  Physical Interactions with 403 interactions from BioGRID | 0.05% |
| --- | --- |
| Liu-Chen-2019  Oncogenic functions of protein kinase D2 and D3 in regulating multiple cancer-related pathways in breast cancer. Liu et al (2019).  *Cancer Med*  Physical Interactions with 106 interactions from BioGRID | 0.05% |
| Liu-Yang-2019  Inflammation-dependent overexpression of c-Myc enhances CRL4<sup>DCAF4</sup> E3 ligase activity and promotes ubiquitination of ST7 in colitis-associated cancer. Liu et al (2019). *J Pathol*  Physical Interactions with 279 interactions from BioGRID | 0.05% |
| Oliviero-Cagney-2015  The variant Polycomb Repressor Complex 1 component PCGF1 interacts with a pluripotency sub-network that includes DPPA4, a regulator of embryogenesis. Oliviero et al (2015). *Sci Rep*  Physical Interactions with 677 interactions from BioGRID | 0.05% |
| Huttlin-Gygi-2015  The BioPlex Network: A Systematic Exploration of the Human Interactome. Huttlin et al (2015). *Cell* Physical Interactions with 23,384 interactions from BioGRID | 0.05% |
| Watanabe-Fujita-2018  GRWD1 regulates ribosomal protein L23 levels via the ubiquitin-proteasome system. Watanabe et al (2018). *J Cell Sci* Physical Interactions with 158 interactions from iRefIndex | 0.05% |
| Hegele-Stelzl-2012 B  Dynamic protein-protein interaction wiring of the human spliceosome. Hegele et al (2012). *Mol Cell* Physical Interactions with 600 interactions from BioGRID | 0.05% |
| So-Colwill-2015  Integrative analysis of kinase networks in TRAIL-induced apoptosis provides a source of potential targets for combination therapy. So et al (2015). *Sci Signal*  Physical Interactions with 652 interactions from BioGRID | 0.05% |
| Conte-Perez-Oliva-2018  USP45 and Spindly are part of the same complex implicated in cell migration. Conte et al (2018). *Sci Rep* Physical Interactions with 161 interactions from iRefIndex | 0.05% |
| Trepte-Wanker-2018 B  LuTHy: a double-readout bioluminescence-based two-hybrid technology for quantitative mapping of protein-protein interactions in mammalian cells. Trepte et al (2018). *Mol Syst Biol*  Physical Interactions with 138 interactions from BioGRID | 0.05% |
| Rual-Vidal-2005  Towards a proteome-scale map of the human protein-protein interaction network. Rual et al (2005). *Nature* | 0.04% |

Rual-Vidal-2005

Physical Interactions with 4,031 interactions from iRefIndex

| Liu-Tan-2018  Proteome-wide analysis of USP14 substrates revealed its role in hepatosteatosis via stabilization of FASN. Liu et al (2018). *Nat Commun*  Physical Interactions with 331 interactions from BioGRID | 0.04% |
| --- | --- |
| IREF-matrixdb  Physical Interactions with 15,422 interactions from iRefIndex | 0.04% |
| Huber-Hoelz-2017  Histone-binding of DPF2 mediates its repressive role in myeloid differentiation. Huber et al (2017). *Proc Natl Acad Sci U S A* Physical Interactions with 217 interactions from iRefIndex | 0.04% |
| Teixeira-Laman-2016 A  Gsk3 and Tomm20 are substrates of the SCFFbxo7/PARK15 ubiquitin ligase associated with Parkinson's disease. Teixeira et al  (2016). *Biochem J*  Physical Interactions with 130 interactions from BioGRID | 0.04% |
| Coyaud-Raught-2015  BioID-based Identification of Skp Cullin F-box (SCF) -TrCP1/2 E3 Ligase Substrates. Coyaud et al (2015). *Mol Cell Proteomics* Physical Interactions with 164 interactions from BioGRID | 0.04% |
| Guard-Old-2019  The nuclear interactome of DYRK1A reveals a functional role in DNA damage repair. Guard et al (2019). *Sci Rep* Physical Interactions with 105 interactions from BioGRID | 0.04% |
| Rengasamy-Walsh-2017  The PRMT5/WDR77 complex regulates alternative splicing through ZNF326 in breast cancer. Rengasamy et al (2017). *Nucleic Acids Res*  Physical Interactions with 103 interactions from iRefIndex | 0.04% |
| Bantscheff-Drewes-2011  Chemoproteomics profiling of HDAC inhibitors reveals selective targeting of HDAC complexes. Bantscheff et al (2011). *Nat Biotechnol*  Physical Interactions with 103 interactions from BioGRID | 0.04% |
| Asadi-Dhanvantari-2018  Plasticity in the Glucagon Interactome Reveals Novel Proteins That Regulate Glucagon Secretion in -TC1-6 Cells. Asadi et al  (2018). *Front Endocrinol (Lausanne)*  Physical Interactions with 229 interactions from BioGRID | 0.04% |
| Giurato-Tarallo-2018  Quantitative mapping of RNA-mediated nuclear estrogen receptor interactome in human breast cancer cells. Giurato et al  (2018). *Sci Data*  Physical Interactions with 2,161 interactions from BioGRID | 0.04% |
| Rivera-Paes Leme-2018  Agrin has a pathological role in the progression of oral cancer. Rivera et al (2018). *Br J Cancer* Physical Interactions with 194 interactions from BioGRID | 0.04% |

0.04%

Yao-Stagljar-2017 B

A Global Analysis of the Receptor Tyrosine Kinase-Protein Phosphatase Interactome. Yao et al (2017). *Mol Cell* Physical Interactions with 325 interactions from BioGRID

| Zeller-Wei-2006  Global mapping of c-Myc binding sites and target gene networks in human B cells. Zeller et al (2006). *Proc Natl Acad Sci U S A* Physical Interactions with 634 interactions from iRefIndex | 0.04% |
| --- | --- |
| Choi-Beutler-2019  LMBR1L regulates lymphopoiesis through Wnt/ -catenin signaling. Choi et al (2019). *Science* Physical Interactions with 928 interactions from BioGRID | 0.04% |
| Pankow-Yates-2015  F508CFTR interactome remodelling promotes rescue of cystic fibrosis. Pankow et al (2015). *Nature* Physical Interactions with 637 interactions from BioGRID | 0.04% |
| Zhang-Zou-2011  A bead-based approach for large-scale identification of in vitro kinase substrates. Zhang et al (2011). *Proteomics* Physical Interactions with 163 interactions from iRefIndex | 0.04% |
| van Wijk-Timmers-2009  A comprehensive framework of E2-RING E3 interactions of the human ubiquitin-proteasome system. van Wijk et al (2009). *Mol Syst Biol*  Physical Interactions with 322 interactions from iRefIndex | 0.04% |
| Scholz-Taylor-2016  FIH Regulates Cellular Metabolism through Hydroxylation of the Deubiquitinase OTUB1. Scholz et al (2016). *PLoS Biol* Physical Interactions with 134 interactions from BioGRID | 0.03% |
| Abbasi-Schild-Poulter-2019  Mapping the Ku Interactome Using Proximity-Dependent Biotin Identification in Human Cells. Abbasi et al (2019). *J Proteome Res*  Physical Interactions with 166 interactions from iRefIndex | 0.03% |
| Pichlmair-Superti-Furga-2011  IFIT1 is an antiviral protein that recognizes 5'-triphosphate RNA. Pichlmair et al (2011). *Nat Immunol* Physical Interactions with 99 interactions from BioGRID | 0.03% |
| Kahle-Zoghbi-2011  Comparison of an expanded ataxia interactome with patient medical records reveals a relationship between macular degeneration and ataxia. Kahle et al (2011). *Hum Mol Genet*  Physical Interactions with 132 interactions from iRefIndex | 0.03% |
| Wilkinson-Coba-2019  Endogenous Cell Type-Specific Disrupted in Schizophrenia 1 Interactomes Reveal Protein Networks Associated With Neurodevelopmental Disorders. Wilkinson et al (2019). *Biol Psychiatry*  Physical Interactions with 100 interactions from iRefIndex | 0.03% |
| Woods-Monteiro-2012 B  Charting the landscape of tandem BRCT domain-mediated protein interactions. Woods et al (2012). *Sci Signal* Physical Interactions with 325 interactions from BioGRID | 0.03% |

Cloutier-Coulombe-2017

0.03%

R2TP/Prefoldin-like component RUVBL1/RUVBL2 directly interacts with ZNHIT2 to regulate assembly of U5 small nuclear ribonucleoprotein. Cloutier et al (2017). *Nat Commun*

Physical Interactions with 506 interactions from BioGRID

| Chen-Guan-2018  SNIP1 Recruits TET2 to Regulate c-MYC Target Genes and Cellular DNA Damage Response. Chen et al (2018). *Cell Rep* Physical Interactions with 240 interactions from BioGRID | 0.03% |
| --- | --- |
| Kumar-Vertegaal-2017  The STUbL RNF4 regulates protein group SUMOylation by targeting the SUMO conjugation machinery. Kumar et al (2017). *Nat Commun*  Physical Interactions with 1,198 interactions from BioGRID | 0.03% |
| Lim-Zoghbi-2006  A protein-protein interaction network for human inherited ataxias and disorders of Purkinje cell degeneration. Lim et al (2006).  *Cell*  Physical Interactions with 5,393 interactions from iRefIndex | 0.03% |
| Foster-Marshall-2013  Proteomic analysis of the NOS2 interactome in human airway epithelial cells. Foster et al (2013). *Nitric Oxide* Physical Interactions with 111 interactions from iRefIndex | 0.03% |
| Xu-Ye-2012  SGTA recognizes a noncanonical ubiquitin-like domain in the Bag6-Ubl4A-Trc35 complex to promote endoplasmic reticulum- associated degradation. Xu et al (2012). *Cell Rep*  Physical Interactions with 189 interactions from iRefIndex | 0.03% |
| Jin-Pawson-2004  Proteomic, functional, and domain-based analysis of in vivo 14-3-3 binding proteins involved in cytoskeletal regulation and cellular organization. Jin et al (2004). *Curr Biol*  Physical Interactions with 247 interactions from iRefIndex | 0.03% |
| Soler-López-Aloy-2011  Interactome mapping suggests new mechanistic details underlying Alzheimer's disease. Soler-López et al (2011). *Genome Res* Physical Interactions with 283 interactions from iRefIndex | 0.02% |
| Rodriguez-von Kriegsheim-2016  Substrate-Trapped Interactors of PHD3 and FIH Cluster in Distinct Signaling Pathways. Rodriguez et al (2016). *Cell Rep* Physical Interactions with 2,035 interactions from BioGRID | 0.02% |
| IREF-spike  Physical Interactions with 20,971 interactions from iRefIndex | 0.02% |
| Liu-Wang-2012  Proteomic identification of common SCF ubiquitin ligase FBXO6-interacting glycoproteins in three kinds of cells. Liu et al (2012).  *J Proteome Res*  Physical Interactions with 593 interactions from BioGRID | 0.02% |
| Chen-Yu-2018  An interactome perturbation framework prioritizes damaging missense mutations for developmental disorders. Chen et al (2018).  *Nat Genet* | 0.02% |

Chen-Yu-2018

Physical Interactions with 404 interactions from BioGRID

| Perez-Hernandez-Yáñez-Mó-2013  The intracellular interactome of tetraspanin-enriched microdomains reveals their function as sorting machineries toward exosomes. Perez-Hernandez et al (2013). *J Biol Chem*  Physical Interactions with 446 interactions from BioGRID | 0.02% |
| --- | --- |
| Sundell-Ivarsson-2018  Proteome-wide analysis of phospho-regulated PDZ domain interactions. Sundell et al (2018). *Mol Syst Biol* Physical Interactions with 129 interactions from iRefIndex | 0.02% |
| Hu-Woods-2019  CTDP1 regulates breast cancer survival and DNA repair through BRCT-specific interactions with FANCI. Hu et al (2019). *Cell Death Discov*  Physical Interactions with 103 interactions from BioGRID | 0.02% |
| Wang-Yang-2011  Toward an understanding of the protein interaction network of the human liver. Wang et al (2011). *Mol Syst Biol* Physical Interactions with 3,408 interactions from BioGRID | 0.02% |
| Varjosalo-Gstaiger-2013 A  The protein interaction landscape of the human CMGC kinase group. Varjosalo et al (2013). *Cell Rep* Physical Interactions with 690 interactions from BioGRID | 0.02% |
| IREF-huri  Physical Interactions with 47,604 interactions from iRefIndex | 0.02% |
| Yachie-Roth-2016  Pooled-matrix protein interaction screens using Barcode Fusion Genetics. Yachie et al (2016). *Mol Syst Biol* Physical Interactions with 671 interactions from BioGRID | 0.02% |
| Olma-Pintard-2009  An interaction network of the mammalian COP9 signalosome identifies Dda1 as a core subunit of multiple Cul4-based E3 ligases. Olma et al (2009). *J Cell Sci*  Physical Interactions with 213 interactions from BioGRID | 0.01% |
| Xie-Zhang-2017  FAF1 phosphorylation by AKT accumulates TGF- type II receptor and drives breast cancer metastasis. Xie et al (2017). *Nat Commun*  Physical Interactions with 227 interactions from BioGRID | 0.01% |
| Zhu-Liu-2018  Deubiquitinating enzyme PSMD14 promotes tumor metastasis through stabilizing SNAIL in human esophageal squamous cell carcinoma. Zhu et al (2018). *Cancer Lett*  Physical Interactions with 287 interactions from iRefIndex | 0.01% |
| Bogachek-Weigel-2014  Sumoylation pathway is required to maintain the basal breast cancer subtype. Bogachek et al (2014). *Cancer Cell* Physical Interactions with 134 interactions from iRefIndex | 0.01% |
| St-Denis-Gingras-2016 | 0.01% |

St-Denis-Gingras-2016

Phenotypic and Interaction Profiling of the Human Phosphatases Identifies Diverse Mitotic Regulators. St-Denis et al (2016). *Cell Rep*

Physical Interactions with 783 interactions from BioGRID

| Taipale-Lindquist-2014  A quantitative chaperone interaction network reveals the architecture of cellular protein homeostasis pathways. Taipale et al  (2014). *Cell*  Physical Interactions with 1,227 interactions from iRefIndex | 0.01% |
| --- | --- |
| Wang-Xiong-2019  Impaired plasma membrane localization of ubiquitin ligase complex underlies 3-M syndrome development. Wang et al (2019). *J Clin Invest*  Physical Interactions with 609 interactions from BioGRID | 0.01% |
| Pichlmair-Superti-Furga-2012  Viral immune modulators perturb the human molecular network by common and unique strategies. Pichlmair et al (2012). *Nature* Physical Interactions with 14 interactions from BioGRID | 0.01% |
| Miyamoto-Sato-Yanagawa-2010  A comprehensive resource of interacting protein regions for refining human transcription factor networks. Miyamoto-Sato et al  (2010). *PLoS One*  Physical Interactions with 934 interactions from iRefIndex | 0.01% |
| Kawahara-Paes Leme-2017  Mass spectrometry-based proteomics revealed Glypican-1 as a novel ADAM17 substrate. Kawahara et al (2017). *J Proteomics* Physical Interactions with 114 interactions from BioGRID | 0.01% |
| Raman-Harper-2015  Systematic proteomics of the VCP-UBXD adaptor network identifies a role for UBXN10 in regulating ciliogenesis. Raman et al  (2015). *Nat Cell Biol*  Physical Interactions with 271 interactions from BioGRID | 0.01% |
| Kumar-Maddika-2017  A Human Tyrosine Phosphatase Interactome Mapped by Proteomic Profiling. Kumar et al (2017). *J Proteome Res* Physical Interactions with 1,863 interactions from BioGRID | 0.01% |
| Ravasi-Hayashizaki-2010  An atlas of combinatorial transcriptional regulation in mouse and man. Ravasi et al (2010). *Cell* Physical Interactions with 658 interactions from BioGRID | 0.01% |
| Li-Wang-2016  Defining the Protein-Protein Interaction Network of the Human Protein Tyrosine Phosphatase Family. Li et al (2016). *Mol Cell Proteomics*  Physical Interactions with 1,476 interactions from BioGRID | 0.01% |
| Blandin-Richard-2013  A human skeletal muscle interactome centered on proteins involved in muscular dystrophies: LGMD interactome. Blandin et al  (2013). *Skelet Muscle*  Physical Interactions with 655 interactions from iRefIndex | 0.01% |
| Fragoza-Yu-2019 | 0.00% |

Fragoza-Yu-2019

Extensive disruption of protein interactions by genetic variants across the allele frequency spectrum in human populations. Fragoza et al (2019). *Nat Commun*

Physical Interactions with 603 interactions from iRefIndex

| Liu-Sun-2019  CCT3 acts upstream of YAP and TFCP2 as a potential target and tumour biomarker in liver cancer. Liu et al (2019). *Cell Death Dis*  Physical Interactions with 561 interactions from BioGRID | 0.00% |
| --- | --- |
| Ouyang-Gill-2009  Direct binding of CoREST1 to SUMO-2/3 contributes to gene-specific repression by the LSD1/CoREST1/HDAC complex. Ouyang et al (2009). *Mol Cell*  Physical Interactions with 105 interactions from BioGRID | 0.00% |
| Albers-Koegl-2005  Automated yeast two-hybrid screening for nuclear receptor-interacting proteins. Albers et al (2005). *Mol Cell Proteomics* Physical Interactions with 289 interactions from iRefIndex | 0.00% |
| Swayampakula-Dedhar-2017  The interactome of metabolic enzyme carbonic anhydrase IX reveals novel roles in tumor cell migration and invadopodia/ MMP14-mediated invasion. Swayampakula et al (2017). *Oncogene*  Physical Interactions with 126 interactions from iRefIndex | 0.00% |
| Sahni-Vidal-2015  Widespread macromolecular interaction perturbations in human genetic disorders. Sahni et al (2015). *Cell* Physical Interactions with 591 interactions from BioGRID | 0.00% |
| IREF-biogrid  Physical Interactions with 176,314 interactions from iRefIndex | 0.00% |
| Cao-Chinnaiyan-2014  The central role of EED in the orchestration of polycomb group complexes. Cao et al (2014). *Nat Commun* Physical Interactions with 1,769 interactions from BioGRID  **Co-expression** | 0.00%  8.01% |
| Wang-Maris-2006  Integrative genomics identifies distinct molecular classes of neuroblastoma and shows that multiple genes are targeted by regional alterations in DNA copy number. Wang et al (2006). *Cancer Res*  Co-expression with 270,388 interactions from GEO | 0.60% |
| Mallon-McKay-2013  StemCellDB: the human pluripotent stem cell database at the National Institutes of Health. Mallon et al (2013). *Stem Cell Res* Co-expression with 602,113 interactions from GEO | 0.51% |
| Roth-Zlotnik-2006  Gene expression analyses reveal molecular relationships among 20 regions of the human CNS. Roth et al (2006). *Neurogenetics* Co-expression with 683,844 interactions from GEO | 0.50% |
| Ramaswamy-Golub-2001  Multiclass cancer diagnosis using tumor gene expression signatures. Ramaswamy et al (2001). *Proc Natl Acad Sci U S A* | 0.48% |

Ramaswamy-Golub-2001

Co-expression with 284,829 interactions from supplementary material

| Innocenti-Brown-2011  Identification, replication, and functional fine-mapping of expression quantitative trait loci in primary human liver tissue. Innocenti et al (2011). *PLoS Genet*  Co-expression with 620,205 interactions from GEO | 0.46% |
| --- | --- |
| Alizadeh-Staudt-2000  Distinct types of diffuse large B-cell lymphoma identified by gene expression profiling. Alizadeh et al (2000). *Nature* Co-expression with 92,360 interactions from supplementary material | 0.46% |
| Dobbin-Giordano-2005  Interlaboratory comparability study of cancer gene expression analysis using oligonucleotide microarrays. Dobbin et al (2005).  *Clin Cancer Res*  Co-expression with 452,322 interactions from GEO | 0.46% |
| Rieger-Chu-2004  Toxicity from radiation therapy associated with abnormal transcriptional responses to DNA damage. Rieger et al (2004). *Proc Natl Acad Sci U S A*  Co-expression with 266,879 interactions from GEO | 0.45% |
| Bild-Nevins-2006 B  Oncogenic pathway signatures in human cancers as a guide to targeted therapies. Bild et al (2006). *Nature* Co-expression with 285,368 interactions from GEO | 0.44% |
| Burington-Shaughnessy-2008  Tumor cell gene expression changes following short-term in vivo exposure to single agent chemotherapeutics are related to survival in multiple myeloma. Burington et al (2008). *Clin Cancer Res*  Co-expression with 295,320 interactions from GEO | 0.44% |
| Boldrick-Relman-2002  Stereotyped and specific gene expression programs in human innate immune responses to bacteria. Boldrick et al (2002). *Proc Natl Acad Sci U S A*  Co-expression with 116,197 interactions from supplementary material | 0.39% |
| Arijs-Rutgeerts-2009  Mucosal gene expression of antimicrobial peptides in inflammatory bowel disease before and after first infliximab treatment. Arijs et al (2009). *PLoS One*  Co-expression with 676,695 interactions from GEO | 0.39% |
| Jiang-de Kok-2017  Omics-based identification of the combined effects of idiosyncratic drugs and inflammatory cytokines on the development of drug- induced liver injury. Jiang et al (2017). *Toxicol Appl Pharmacol*  Co-expression with 444,959 interactions from GEO | 0.36% |
| Perou-Botstein-2000  Molecular portraits of human breast tumours. Perou et al (2000). *Nature* Co-expression with 189,373 interactions from supplementary material | 0.36% |
| Chen-Brown-2002  Gene expression patterns in human liver cancers. Chen et al (2002). *Mol Biol Cell* | 0.35% |

| Chen-Brown-2002  Co-expression with 291,300 interactions from supplementary material | |
| --- | --- |
| Wang-Cheung-2015  Genetic variation in insulin-induced kinase signaling. Wang et al (2015). *Mol Syst Biol* Co-expression with 422,896 interactions from GEO | 0.34% |
| Wu-Garvey-2007  The effect of insulin on expression of genes and biochemical pathways in human skeletal muscle. Wu et al (2007). *Endocrine* Co-expression with 275,155 interactions from GEO | 0.28% |
| Rosenwald-Staudt-2001  Relation of gene expression phenotype to immunoglobulin mutation genotype in B cell chronic lymphocytic leukemia. Rosenwald et al (2001). *J Exp Med*  Co-expression with 118,097 interactions from supplementary material | 0.28% |
| Ross-Perou-2001  A comparison of gene expression signatures from breast tumors and breast tissue derived cell lines. Ross et al (2001). *Dis Markers* Co-expression with 146,858 interactions from supplementary material | 0.26% |
| Perou-Botstein-1999  Distinctive gene expression patterns in human mammary epithelial cells and breast cancers. Perou et al (1999). *Proc Natl Acad Sci U S A*  Co-expression with 68,200 interactions from supplementary material  **Predicted** | 0.22%  5.37% |
| I2D-Li-Vidal-2004-CE-DATA-Worm2Human  A map of the interactome network of the metazoan C. elegans. Li et al (2004). *Science* Predicted with 48 interactions from I2D | 0.73% |
| I2D-BioGRID-Yeast2Human  BioGRID: a general repository for interaction datasets. Stark et al (2006). *Nucleic Acids Res* Predicted with 17,314 interactions from I2D | 0.71% |
| I2D-vonMering-Bork-2002-High-Yeast2Human  Comparative assessment of large-scale data sets of protein-protein interactions. von Mering et al (2002). *Nature* Predicted with 723 interactions from I2D | 0.60% |
| I2D-Li-Vidal-2004-interolog-Worm2Human  A map of the interactome network of the metazoan C. elegans. Li et al (2004). *Science* Predicted with 396 interactions from I2D | 0.51% |
| I2D-vonMering-Bork-2002-Medium-Yeast2Human  Comparative assessment of large-scale data sets of protein-protein interactions. von Mering et al (2002). *Nature* Predicted with 1,280 interactions from I2D | 0.40% |
| I2D-Tarassov-PCA-Yeast2Human  An in vivo map of the yeast protein interactome. Tarassov et al (2008). *Science* Predicted with 235 interactions from I2D | 0.29% |
| Wu-Stein-2010 | 0.24% |

| Wu-Stein-2010  A human functional protein interaction network and its application to cancer data analysis. Wu et al (2010). *Genome Biol* Predicted with 89,967 interactions from supplementary material | |
| --- | --- |
| I2D-vonMering-Bork-2002-Low-Yeast2Human  Comparative assessment of large-scale data sets of protein-protein interactions. von Mering et al (2002). *Nature* Predicted with 7,979 interactions from I2D | 0.18% |
| I2D-Wang-Orkin-2006-EScmplxIP-Mouse2Human  A protein interaction network for pluripotency of embryonic stem cells. Wang et al (2006). *Nature* Predicted with 5 interactions from I2D | 0.18% |
| I2D-IntAct-Mouse2Human  The IntAct molecular interaction database in 2010. Aranda et al (2010). *Nucleic Acids Res* Predicted with 11,478 interactions from I2D | 0.16% |
| I2D-INNATEDB-Mouse2Human  InnateDB: facilitating systems-level analyses of the mammalian innate immune response. Lynn et al (2008). *Mol Syst Biol* Predicted with 4,049 interactions from I2D | 0.15% |
| I2D-BioGRID-Rat2Human  BioGRID: a general repository for interaction datasets. Stark et al (2006). *Nucleic Acids Res* Predicted with 2,148 interactions from I2D | 0.14% |
| I2D-BioGRID-Mouse2Human  BioGRID: a general repository for interaction datasets. Stark et al (2006). *Nucleic Acids Res* Predicted with 10,524 interactions from I2D | 0.14% |
| Stuart-Kim-2003  A gene-coexpression network for global discovery of conserved genetic modules. Stuart et al (2003). *Science* Predicted with 25,001 interactions from supplementary material | 0.14% |
| I2D-Yu-Vidal-2008-GoldStd-Yeast2Human  High-quality binary protein interaction map of the yeast interactome network. Yu et al (2008). *Science* Predicted with 173 interactions from I2D | 0.12% |
| I2D-Krogan-Greenblatt-2006-Core-Yeast2Human  Global landscape of protein complexes in the yeast Saccharomyces cerevisiae. Krogan et al (2006). *Nature* Predicted with 860 interactions from I2D | 0.10% |
| I2D-BioGRID-Fly2Human  BioGRID: a general repository for interaction datasets. Stark et al (2006). *Nucleic Acids Res* Predicted with 8,676 interactions from I2D | 0.09% |
| I2D-BIND-Rat2Human  BIND--a data specification for storing and describing biomolecular interactions, molecular complexes and pathways. Bader et al  (2000). *Bioinformatics*  Predicted with 468 interactions from I2D | 0.08% |
| I2D-IntAct-Yeast2Human  The IntAct molecular interaction database in 2010. Aranda et al (2010). *Nucleic Acids Res* | 0.08% |

| I2D-IntAct-Yeast2Human  Predicted with 7,325 interactions from I2D | |
| --- | --- |
| I2D-IntAct-Rat2Human  The IntAct molecular interaction database in 2010. Aranda et al (2010). *Nucleic Acids Res* Predicted with 1,690 interactions from I2D | 0.08% |
| I2D-Chen-Pawson-2009-PiwiScreen-Mouse2Human  Mouse Piwi interactome identifies binding mechanism of Tdrkh Tudor domain to arginine methylated Miwi. Chen et al (2009).  *Proc Natl Acad Sci U S A*  Predicted with 29 interactions from I2D | 0.06% |
| I2D-BIND-Mouse2Human  BIND--a data specification for storing and describing biomolecular interactions, molecular complexes and pathways. Bader et al  (2000). *Bioinformatics*  Predicted with 1,007 interactions from I2D | 0.05% |
| I2D-Krogan-Greenblatt-2006-NonCore-Yeast2Human  Global landscape of protein complexes in the yeast Saccharomyces cerevisiae. Krogan et al (2006). *Nature* Predicted with 678 interactions from I2D | 0.04% |
| I2D-MGI-Mouse2Human  Ontological visualization of protein-protein interactions. Drabkin et al (2005). *BMC Bioinformatics* Predicted with 595 interactions from I2D | 0.04% |
| I2D-MINT-Rat2Human  MINT: a Molecular INTeraction database. Zanzoni et al (2002). *FEBS Lett* Predicted with 540 interactions from I2D | 0.04% |
| I2D-BIND-Worm2Human  BIND--a data specification for storing and describing biomolecular interactions, molecular complexes and pathways. Bader et al  (2000). *Bioinformatics*  Predicted with 349 interactions from I2D | 0.02% |
| I2D-BIND-Yeast2Human  BIND--a data specification for storing and describing biomolecular interactions, molecular complexes and pathways. Bader et al  (2000). *Bioinformatics*  Predicted with 599 interactions from I2D | 0.01% |
| I2D-Formstecher-Daviet-2005-Embryo-Fly2Human  Protein interaction mapping: a Drosophila case study. Formstecher et al (2005). *Genome Res* Predicted with 270 interactions from I2D  **Co-localization** | 0.00%  3.63% |
| Zhang-Shang-2006  The catalytic subunit of the proteasome is engaged in the entire process of estrogen receptor-regulated transcription. Zhang et al  (2006). *EMBO J*  Co-localization with 53 interactions from BioGRID | 1.44% |

Schadt-Shoemaker-2004

A comprehensive transcript index of the human genome generated using microarrays and computational approaches. Schadt et al

(2004). *Genome Biol*

1.11%

**Co-localization** 3.63%

| Schadt-Shoemaker-2004  Co-localization with 59,920 interactions from GEO | |
| --- | --- |
| Johnson-Shoemaker-2003  Genome-wide survey of human alternative pre-mRNA splicing with exon junction microarrays. Johnson et al (2003). *Science* Co-localization with 426,464 interactions from GEO | 0.71% |
| Chen-Huang-2014  Using an in situ proximity ligation assay to systematically profile endogenous protein-protein interactions in a pathway network. Chen et al (2014). *J Proteome Res*  Co-localization with 559 interactions from BioGRID  **Genetic Interactions** | 0.37%  2.87% |
| Shen-Mali-2017  Combinatorial CRISPR-Cas9 screens for de novo mapping of genetic interactions. Shen et al (2017). *Nat Methods* Genetic Interactions with 152 interactions from BioGRID | 0.32% |
| Toyoshima-Grandori-2012  Functional genomics identifies therapeutic targets for MYC-driven cancer. Toyoshima et al (2012). *Proc Natl Acad Sci U S A* Genetic Interactions with 101 interactions from BioGRID | 0.27% |
| Matsuoka-Elledge-2007  ATM and ATR substrate analysis reveals extensive protein networks responsive to DNA damage. Matsuoka et al (2007). *Science* Genetic Interactions with 342 interactions from iRefIndex | 0.25% |
| Vizeacoumar-Moffat-2013  A negative genetic interaction map in isogenic cancer cell lines reveals cancer cell vulnerabilities. Vizeacoumar et al (2013). *Mol Syst Biol*  Genetic Interactions with 201 interactions from BioGRID | 0.25% |
| Xie-Green-2012  A synthetic interaction screen identifies factors selectively required for proliferation and TERT transcription in p53-deficient human cancer cells. Xie et al (2012). *PLoS Genet*  Genetic Interactions with 100 interactions from BioGRID | 0.23% |
| Srivas-Ideker-2016  A Network of Conserved Synthetic Lethal Interactions for Exploration of Precision Cancer Therapy. Srivas et al (2016). *Mol Cell* Genetic Interactions with 173 interactions from BioGRID | 0.23% |
| BIOGRID-SMALL-SCALE-STUDIES Genetic Interactions with 651 interactions from BioGRID | 0.22% |
| IREF-SMALL-SCALE-STUDIES  Genetic Interactions with 1,159 interactions from iRefIndex | 0.22% |
| Han-Bassik-2017 A  Synergistic drug combinations for cancer identified in a CRISPR screen for pairwise genetic interactions. Han et al (2017). *Nat Biotechnol*  Genetic Interactions with 1,042 interactions from BioGRID | 0.22% |
| Du-Krogan-2017 | 0.11% |

Du-Krogan-2017

Genetic interaction mapping in mammalian cells using CRISPR interference. Du et al (2017). *Nat Methods* Genetic Interactions with 166 interactions from BioGRID

| Horlbeck-Gilbert-2018 A  Mapping the Genetic Landscape of Human Cells. Horlbeck et al (2018). *Cell* Genetic Interactions with 1,808 interactions from BioGRID | 0.11% |
| --- | --- |
| Horlbeck-Gilbert-2018 B  Mapping the Genetic Landscape of Human Cells. Horlbeck et al (2018). *Cell* Genetic Interactions with 946 interactions from BioGRID | 0.10% |
| Blomen-Brummelkamp-2015  Gene essentiality and synthetic lethality in haploid human cells. Blomen et al (2015). *Science* Genetic Interactions with 127 interactions from BioGRID | 0.09% |
| Han-Bassik-2017 B  Synergistic drug combinations for cancer identified in a CRISPR screen for pairwise genetic interactions. Han et al (2017). *Nat Biotechnol*  Genetic Interactions with 893 interactions from BioGRID | 0.07% |
| Willingham-Muchowski-2003  Yeast genes that enhance the toxicity of a mutant huntingtin fragment or alpha-synuclein. Willingham et al (2003). *Science* Genetic Interactions with 37 interactions from BioGRID | 0.06% |
| Xiao-Brown-2018  Estrogen-regulated feedback loop limits the efficacy of estrogen receptor-targeted breast cancer therapy. Xiao et al (2018). *Proc Natl Acad Sci U S A*  Genetic Interactions with 684 interactions from BioGRID | 0.06% |
| Achuthankutty-Mailand-2019  Regulation of ETAA1-mediated ATR activation couples DNA replication fidelity and genome stability. Achuthankutty et al  (2019). *J Cell Biol*  Genetic Interactions with 99 interactions from BioGRID | 0.03% |
| Luo-Elledge-2009  A genome-wide RNAi screen identifies multiple synthetic lethal interactions with the Ras oncogene. Luo et al (2009). *Cell* Genetic Interactions with 316 interactions from BioGRID | 0.03% |
| Lin-Smith-2010  A genome-wide map of human genetic interactions inferred from radiation hybrid genotypes. Lin et al (2010). *Genome Res* Genetic Interactions with 4,805,334 interactions from supplementary material  **Pathway** | 0.01%  1.88% |
| Wu-Stein-2010  A human functional protein interaction network and its application to cancer data analysis. Wu et al (2010). *Genome Biol* Pathway with 78,117 interactions from supplementary material | 0.60% |
| NCI NATURE  Pathway with 10,118 interactions from Pathway Commons | 0.39% |

**Pathway** 1.88%

| REACTOME  Pathway with 24,890 interactions from Pathway Commons | 0.38% |
| --- | --- |
| IMID  Pathway with 1,023 interactions from Pathway Commons | 0.27% |
| CELL MAP  Pathway with 397 interactions from Pathway Commons | 0.23% |
| HUMANCYC  Pathway with 681 interactions from Pathway Commons  **Shared protein domains** | 0.01%  0.60% |
| INTERPRO  Shared protein domains with 621,159 interactions from InterPro | 0.39% |

PFAM

Shared protein domains with 471,533 interactions from Pfam
